# The Acetate/ACSS2 Switch Regulates HIF-2 Stress Signaling in the Tumor Cell Microenvironment

**DOI:** 10.1371/journal.pone.0116515

**Published:** 2015-02-17

**Authors:** Rui Chen, Min Xu, Jason S. Nagati, Richard T. Hogg, Alok Das, Robert D. Gerard, Joseph A. Garcia

**Affiliations:** 1 Department of Internal Medicine, University of Texas Southwestern Medical Center, Dallas, Texas, United States of America; 2 Department of Molecular Biology, University of Texas Southwestern Medical Center, Dallas, Texas, United States of America; 3 Key Laboratory of Environmental Medicine Engineering, Ministry of Education, School of Public Health, Southeast University, Nanjing, China; 4 Department of Medicine, Veterans Affairs North Texas Health Care System, Dallas, Texas, United States of America; University of Dundee, UNITED KINGDOM

## Abstract

Optimal stress signaling by Hypoxia Inducible Factor 2 (HIF-2) during low oxygen states or hypoxia requires coupled actions of a specific coactivator/lysine acetyltransferase, Creb binding protein (CBP), and a specific deacetylase, Sirtuin 1 (SIRT1). We recently reported that acetylation of HIF-2 by CBP also requires a specific acetyl CoA generator, acetate-dependent acetyl CoA synthetase 2 (ACSS2). In this study, we demonstrate that ACSS2/HIF-2 signaling is active not only during hypoxia, but also during glucose deprivation. Acetate levels increase during stress and coincide with maximal HIF-2α acetylation and CBP/HIF-2α complex formation. Exogenous acetate induces HIF-2α acetylation, CBP/HIF-2α complex formation, and HIF-2 signaling. ACSS2 and HIF-2 are required for maximal colony formation, proliferation, migration, and invasion during stress. Acetate also stimulates flank tumor growth and metastasis in mice in an ACSS2 and HIF-2 dependent manner. Thus, ACSS2/CBP/SIRT1/HIF-2 signaling links nutrient sensing and stress signaling with cancer growth and progression in mammals.

## Introduction

The tumor microenvironment of solid tumors is characterized by oxygen and glucose deficits. To survive, tumor cells sense and respond to various environmental stresses by activating specific stress-responsive signal transducers. Hypoxia Inducible Factor (HIF) are heterodimeric transcription factors comprised of one of three regulated alpha subunits and a shared beta subunit [1]. HIF family diversity may allow cells to survive the pleiotropic environmental stresses encountered *in vivo*. HIFs are frequently active in cancer and can promote cell survival, making HIFs attractive targets for cancer therapies. However, because HIF-1 and HIF-2 can have opposing roles in cancer [2,3,4], indiscriminant HIF inhibition may not improve outcomes.

HIF-1 transactivation is controlled by changes in HIF-1α degradation rates, which is regulated by oxygen-dependent prolyl hydroxylases, and by p300/CBP coactivator recruitment to the HIF-1α carboxy terminal activation domain (CTAD), which is regulated by the oxygen-requiring asparaginyl hydroxylase Factor Inhibiting HIF-1 (FIH1) [5,6,7]. Although HIF-2α is closely related to HIF-1α, HIF-2 signaling is prominently regulated by acetylation and deacetylation, post-translational modifications that are not strictly oxygen-dependent. Acetylation and deacetylation of HIF-2α is conferred by CBP [8] and SIRT1 [9], respectively. Rather than functioning as a binary on/off switch, acetylation and deacetylation are both required for stable CBP/HIF-2α complex formation and maximal HIF-2 signaling during hypoxia. Acetylation of HIF-2α by CBP is rate-limiting and is regulated by the production of acetyl CoA generated by a selective acetyl CoA generator, acetyl CoA synthetase 2 (ACSS2) [10].

A thorough understanding of how HIF-1 and HIF-2 signal transduction is mediated may provide clues as to how selective inhibition of each HIF member might be accomplished. For example, differences have been noted between HIF-1 and HIF-2 with respect to the response to stresses besides hypoxia. During glucose deprivation, HIF-2α, but not HIF-1α, controls the proliferative response of embryonic stem cells [11] and is stabilized in Ewing’s sarcoma cells [12]. During combined oxygen/glucose deprivation, HIF-1α, but not HIF-2α, levels decrease in HT1080 cells [13]. Similar to what occurs during hypoxia, we hypothesize that glucose deprivation activates HIF-2, but not HIF-1, signaling by inducing ACSS2-dependent CBP/HIF-2α interactions.

Linking changes in cellular metabolism to signal transduction via stress-dependent post-translational modifications of specific signal transducers allows for a prompt response to environmental stress at the gene expression level. We reasoned that acetylation/deacetylation confer dynamic responsiveness of HIF-2 signaling to the environmental stresses of hypoxia and glucose deprivation experienced by solid tumors. Using molecular and biochemical assays, we examined the temporal aspects of and mechanistic basis for acetylation and coactivator complex formation in HIF-2 signaling during hypoxia or glucose deprivation for HT1080 cells, a fibrosarcoma-derived cell line in which CBP-mediated acetylation and SIRT1-regulated deacetylation of HIF-2α occurs [14]. Finally, to determine the biological significance of these findings, we utilized relevant cell culture and mouse models of cancer growth and development.

## Results

### Acetate controls CBP/HIF-2α interactions during stress

**Fig 1:**
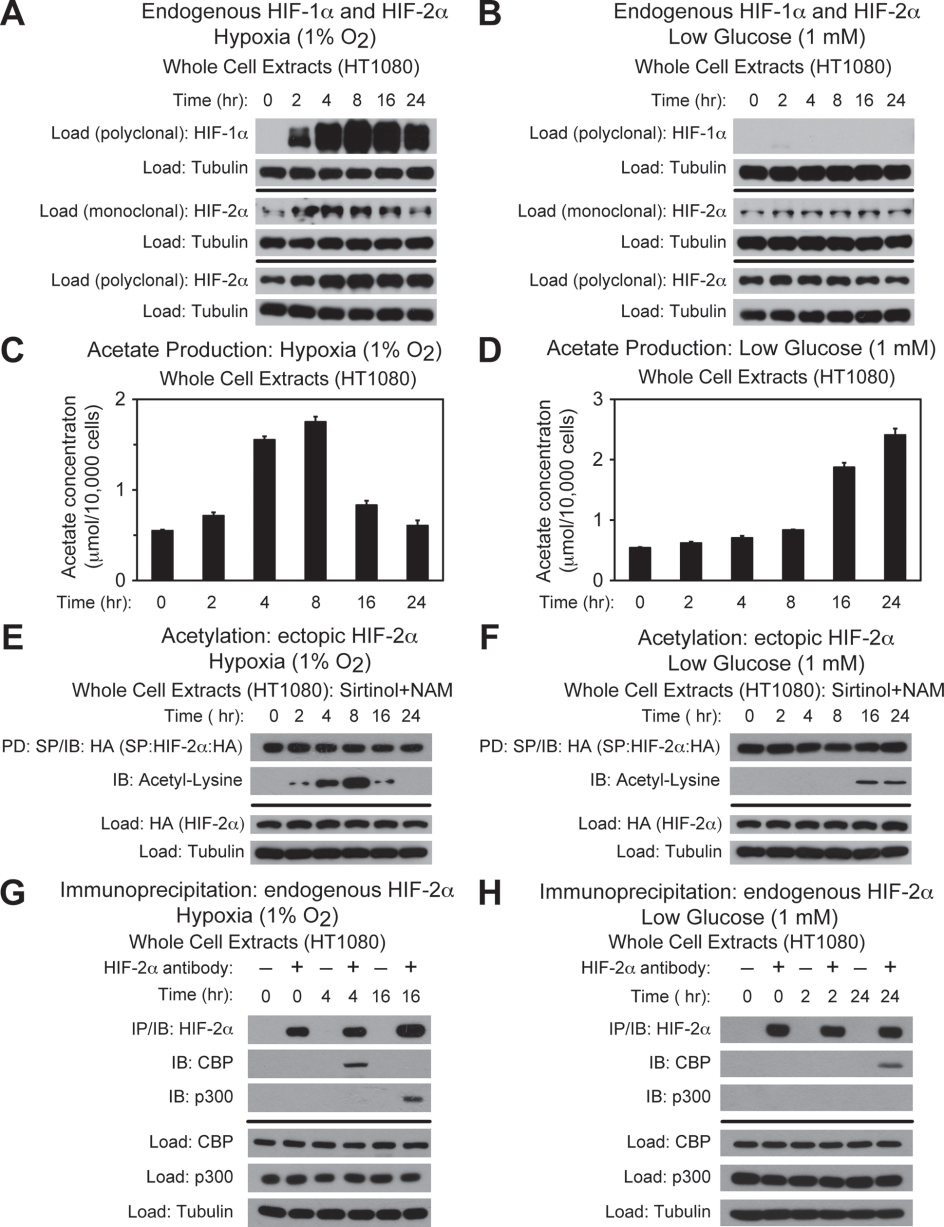
Acetate controls CBP/HIF-2α interactions during stress. (A) Detection of endogenous HIF-1α or HIF-2α in whole cell extracts by immunoblotting after the indicated period of hypoxia exposure. Alpha-tubulin levels for each immunoblot are also shown. (B) Same as (A) except after low glucose exposure. (C) Cellular acetate levels generated after the indicated period of hypoxia exposure (*n* = 3 biological replicates/time-point; single measurement/replicate; mean/SD). (D) Same as (C) except after low glucose exposure. (E) Acetylation of ectopic HA-tagged HIF-2α after pulldown (PD) and detection by immunoblotting (IB) after the indicated period of hypoxia exposure with pharmacological inhibition of Sirt1 by sirtinol and nicotinamide (NAM). Levels of ectopic HIF-2α and alpha-tubulin levels from whole cell extracts prepared under the same conditions are also shown. (F) Same as (E) except after low glucose exposure. (G) Detection of endogenous CBP/HIF-2α or p300/HIF-2α complexes by immunoblotting (IB) after early (4 hr) or late (16 hr) hypoxia exposure. (H) Same as (G) except after early (2 hr) or late (24 hr) low glucose exposure. All experiments performed with HT1080 whole cell extracts.

Because the tumor microenvironment is frequently characterized by deficiencies in oxygen and glucose content, we first asked whether HIF-1α and HIF-2α protein levels vary in HT1080 cells, a rapidly growing cancer cell line derived from a human fibrosarcoma, subjected to hypoxia or glucose deprivation. During hypoxia, HIF-1α protein levels increase markedly; in comparison, HIF-2α protein levels, detected either by mouse monoclonal or rabbit polyclonal antibody, are only modestly increased (Fig. 1A). During glucose deprivation, HIF-1α levels are virtually undetectable and HIF-2α protein levels are unchanged compared to normal conditions (Fig. 1B). Thus, HIF-1α and HIF-2α protein levels in HT1080 cells differ with respect to basal as well as stress-induced relative levels.

The modest change in HIF-2α levels during hypoxia raises the possibility that other factors besides increases in protein mass are important for HIF-2 signaling. We recently demonstrated that acetate-dependent acetylation of HIF-2α is important for HIF-2 signaling in Hep3B cells induced by hypoxia and in mice with HIF-2 signaling induced by anemia [10]. We therefore asked whether hypoxia or glucose deprivation results in acetate generation in HT1080 cells. Following hypoxia exposure, acetate increases and peaks at 4 to 8 hr (Fig. 1C). Glucose deprivation also results in acetate production (Fig. 1D), although the kinetics between hypoxia and glucose deprivation differ with acetate levels peaking at later times during glucose deprivation (16 and 24 hr).

The lysine acetyltransferase/coactivator CBP regulates two coupled processes, HIF-2α acetylation and CBP/HIF-2α complex formation, in Hep3B cells exposed to hypoxia [8]. We next asked if these same processes parallel acetate levels in HT1080 cells exposed to hypoxia or glucose deprivation as occurs in Hep3B cells exposed to hypoxia [10]. Acetylation of ectopic HIF-2α mirrors acetate production and peaks at the same times when acetate levels are maximal for both hypoxia and glucose deprivation (Fig. 1E-F). In addition, CBP/HIF-2α complex formation also is evident at times when acetate production and HIF-2α acetylation are maximal (Fig. 1G-H), consistent with acetylation of HIF-2α by CBP providing a molecular platform for stable CBP/HIF-2α complex formation [8]. Of note, stable p300/HIF-2α complex formation is only evident for late hypoxia, but is not evident for HT1080 cells exposed to either early or late glucose deprivation. Thus, CBP-mediated biochemical and molecular interactions occur during hypoxia as well as during glucose deprivation and mirror cellular acetate levels, although the timing of peak acetate generation differs in each of these two stresses. Furthermore, the absence of p300/HIF-2α complexes during glucose deprivation suggests that p300/HIF-2α complex formation is an oxygen-dependent and acetate-independent process.

### ACSS2 is the molecular mediator of the acetate switch

**Fig 2:**
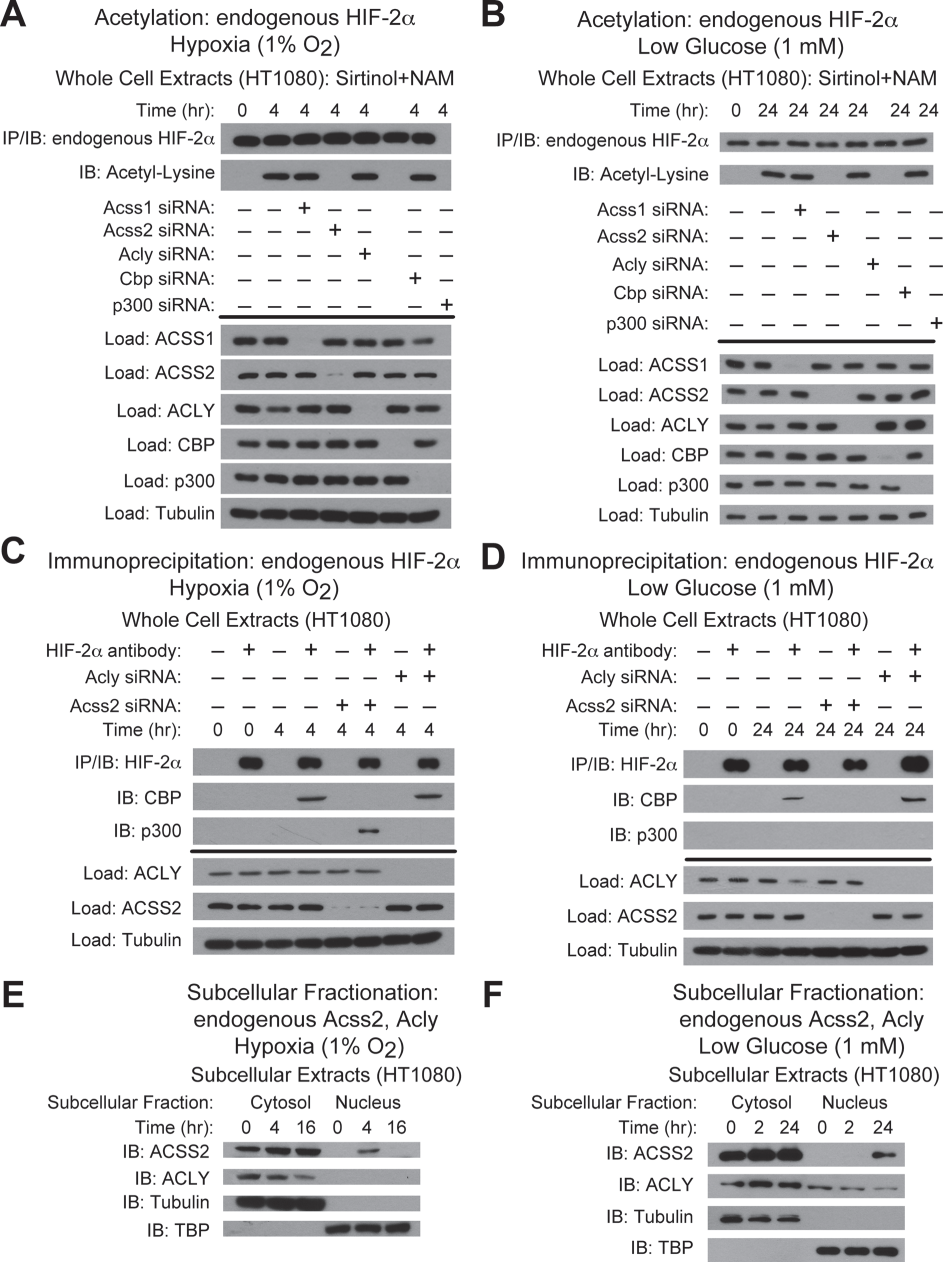
ACSS2 is the molecular mediator of the acetate switch. (A) Acetylation of endogenous HIF-2α after immunoprecipitation (IP) and detection by immunoblotting (IB) after early (4 hr) hypoxia exposure with pharmacological inhibition of Sirt1 by sirtinol and nicotinamide (NAM). (B) Same as (A) except after late (24 hr) low glucose exposure. (C) Detection of endogenous CBP/HIF-2α or p300/HIF-2α complexes by immunoblotting (IB) after early (4 hr) hypoxia exposure. (D) Same as (C) except after late (24 hr) low glucose exposure. (E) Detection of ACSS2 or ACLY in HT1080 subcellular fractions by immunoblotting (IB) after early (4 hr) or late (16 hr) hypoxia exposure. (F) Same as (E) except after early (2 hr) or late (24 hr) low glucose exposure. Experiments in (A)-(D) performed with HT1080 whole cell extracts.

We next sought to determine whether the acetyl CoA generators ACSS1, ACSS2, and ACLY are required for acetylation of endogenous HIF-2α using siRNA knockdown as we did previously in Hep3B cells [10]. For HT1080 cells exposed either to hypoxia or glucose deprivation, HIF-2α acetylation requires ACSS2 and CBP (Fig. 2A-B). ACSS2 is also required for stable CBP/HIF-2α complex formation during hypoxia and glucose deprivation (Fig. 2C-D). Thus, ACSS2 is required for CBP/HIF-2α biochemical and molecular interactions induced in HT1080 cells following exposure to low oxygen or glucose states. Because a fraction of ACSS2 localizes to the nucleus in Hep3B cells exposed to hypoxia [10], we asked if ACSS2 in HT1080 cells changes subcellular localization in response to hypoxia or glucose deprivation. Indeed, a subset of ACSS2, but not ACLY, transits to the nucleus during hypoxia or glucose deprivation at times that coincide with maximal acetate production (Fig. 2E-F), which suggests that acetate is a biochemical trigger for this event.

### Acetate induces ACSS2-dependent CBP/HIF-2α interactions

**Fig 3:**
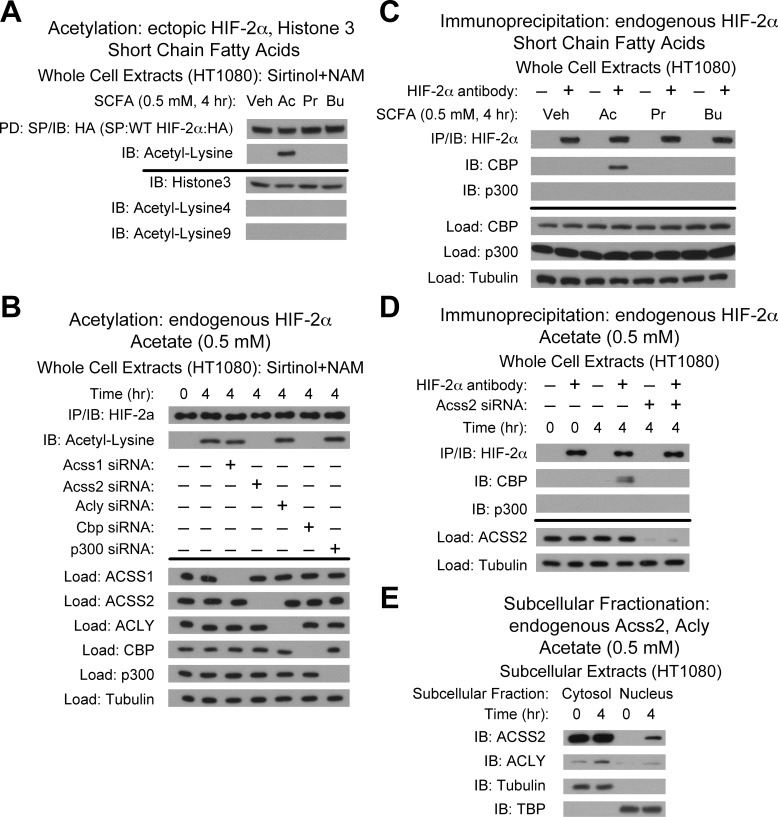
Acetate induces ACSS2-dependent CBP/HIF-2α interactions. (A) Acetylation of ectopic HA-tagged wild-type HIF-2α after pulldown (PD) and detection by immunoblotting (IB) after (4 hr) incubation with vehicle (Veh), or the short chain fatty acids acetate (Ac), propionate (Pr), and butyrate (Bu) with pharmacological inhibition of Sirt1 by sirtinol and nicotinamide (NAM). (B) Acetylation of endogenous HIF-2α after immunoprecipitation (IP) and detection by immunoblotting (IB) after (4 hr) incubation with acetate and following control, ACSS1, ACSS2, ACLY, CBP or p300 knockdown. (C) Endogenous CBP/HIF-2α or p300/HIF-2α complexes after (4 hr) treatment with vehicle (Veh), acetate (Ac), propionate (Pr), or butyrate (Bu). (D) Endogenous CBP/HIF-2α or p300/HIF-2α complexes detected by immunoblotting (IB) after (4 hr) acetate exposure following ACSS2 knockdown. (E) Detection of ACSS2 or ACLY in HT1080 subcellular fractions by immunoblotting (IB) after (4 hr) acetate exposure. Experiments in (A)-(D) performed with HT1080 whole cell extracts.

As observed with Hep3B cells [10], conditioned media from hypoxia-exposed HT1080 cells at peak acetate generation induces endogenous HIF-2α acetylation (S1A Fig.). Conditioned media obtained at peak acetate generation for HT1080 cells maintained under low glucose conditions also induces endogenous HIF-2α acetylation (S1B Fig.). Similar to Hep3B cells [10], the addition of acetate, but not other short chain fatty acids (SCFA), to HT1080 cells directly induces acetylation of ectopic HIF-2α (Fig. 3A), a process that requires ACSS2 and CBP (Fig. 3B), but does not induce acetylation of histone 3 protein. Addition of acetate also induces stable CBP/HIF-2α complex formation (Fig. 3C), which requires ACSS2 (Fig. 3D), and induces nuclear translocation of ACSS2 but not ACLY (Fig. 3E). Thus, acetate is a specific SCFA that triggers ACSS2-dependent biochemical and molecular interactions of CBP with HIF-2α in HT1080 cells exposed to hypoxia or glucose deprivation.

Similar to results observed in Hep3B cells exposed to hypoxia or exogenous acetate [8], stable CBP/HIF-2α complexes form in HT1080 cells following hypoxia, glucose deprivation, or acetate exposure only if SIRT1 and CBP are present (S2A-C Fig.). Thus, stable CBP/HIF-2α complex formation in HT1080 cells requires not only the acetate/ACSS2 switch, but also the coupled actions of CBP and SIRT1. We asked if the oxygen-dependent asparagine hydroxylase Factor Inhibiting HIF-1 (FIH1), which regulates p300 recruitment to the HIF-1α CTAD under hypoxia [5,7], modulates CBP or p300 recruitment to HIF-2α in HT1080 cells during hypoxia or glucose deprivation. FIH1 knockdown results in CBP/HIF-2α and p300/HIF-2α complex formation during early (4 hr) hypoxia; ACSS2 knockdown results exclusively in p300/HIF-2α complex formation under these same conditions with or without FIH1 (S2D Fig.). In comparison, p300/HIF-2α complex formation under glucose deprivation or following acetate addition occurs only with FIH1 knockdown, but is not evident following ACSS2 knockdown (S2E-F Fig.), indicating that the determinants for CTAD-independent p300/HIF-2α complex formation differ between hypoxia and glucose deprivation.

We next defined the *in vitro* determinants of CBP/HIF-2α complex formation using extracts derived from HT1080 cells exposed to hypoxia or to low glucose conditions. Supplementing ACSS2-depleted extracts obtained at early (4 hr) hypoxia (S3A Fig.) or late (16 hr) low glucose (S3C Fig.) time-points with acetyl CoA results in the formation of CBP/HIF-2α complexes. Furthermore, CBP/HIF-2α complex formation in ACSS2-replete late (16 hr) hypoxia extracts requires both acetate and ATP (S3B Fig.), whereas CBP/HIF-2α complex formation with ACSS2-replete early (2 hr) low glucose extracts requires only acetate (S3D Fig.). Hence, hypoxia and glucose deprivation have unique effects on the kinetics associated with production of acetate, a substrate required by ACSS2 for acetyl CoA generation, as well as of ATP, a co-factor also required for ACSS2 enzymatic action [15].

### ACSS2, CBP, and SIRT1 are required for HIF-2 signaling

**Fig 4:**
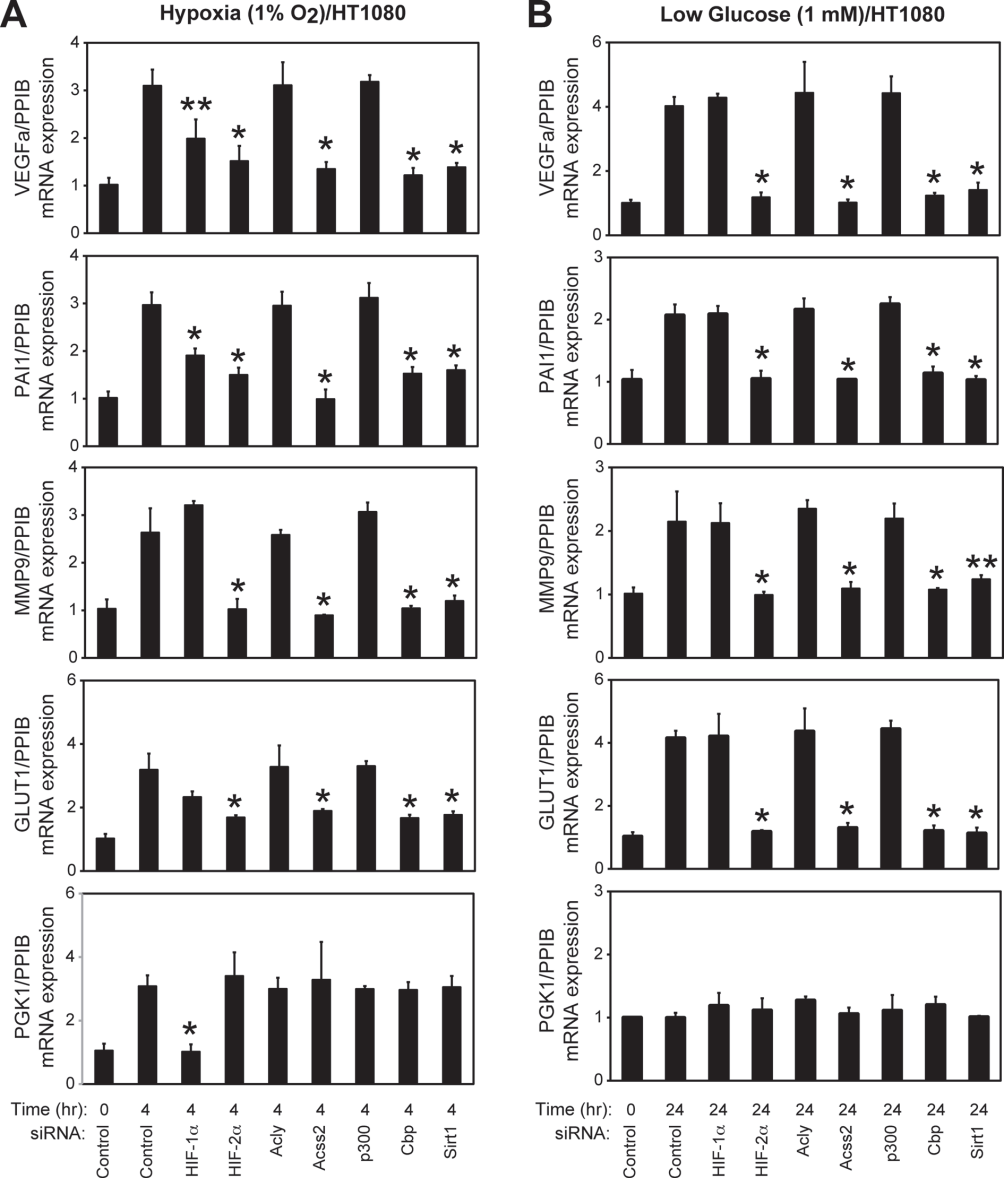
ACSS2, CBP, and SIRT1 are required for HIF-2 signaling. Semi-quantitative RTPCR measurement of HIF-1 selective (PGK1), HIF-2 selective (MMP9, GLUT1), or HIF-1/HIF-2 co-regulated (VEGFa, PAI1) target genes following HIF-1α, HIF-2α, ACLY, ACSS2, p300, CBP, or SIRT1 knockdown and after (A) early (4 hr) hypoxia exposure, or (B) after incubation under late (24 hr) low glucose conditions. Comparison by one-tailed t-test between control knockdown/treatment and specified knockdown/treatment with significant reductions compared to control indicated (single pool of triplicate biological replicates/manipulation; triplicate measurements/pool; mean/SD; *, P<0.05; **, P<0.10).

Using siRNA knockdown in HT1080 cells, we examined the role of HIFs (HIF-1 and HIF-2), acetyl CoA generators (ACLY and ACSS2), acetylases (p300 and CBP), and a deacetylase (SIRT1) in HIF target gene induction under stress conditions (Fig. 4A-B). Genes induced in part (*VEGFA*, *PAI1*) or preferentially by HIF-2 (*MMP9*, *GLUT1*) under hypoxia are affected by ACSS2, CBP, and SIRT1 knockdown whereas a HIF-1 target gene (*PGK1)* is not affected by knockdown of any of these factors (Fig. 4A). ACLY knockdown has no effect on HIF target gene induction. Furthermore, HIF-2 target gene induction during glucose deprivation is blunted by ACSS2, CBP, and SIRT1 knockdown (Fig. 4B). There is no induction of *PGK1* during low glucose, consistent with the absence of HIF-1α protein [13]. Thus, when activated by tumor-associated environmental stresses, the acetate/ACSS2 switch acts in conjunction with CBP, SIRT1, and HIF-2 to form a signaling axis defined and united by molecular and biochemical interactions, which ultimately regulate expression of target genes associated with growth as well as survival of tumor cells.

### ACSS2/CBP mediate acetate augmentation of HIF-2 signaling

**Fig 5:**
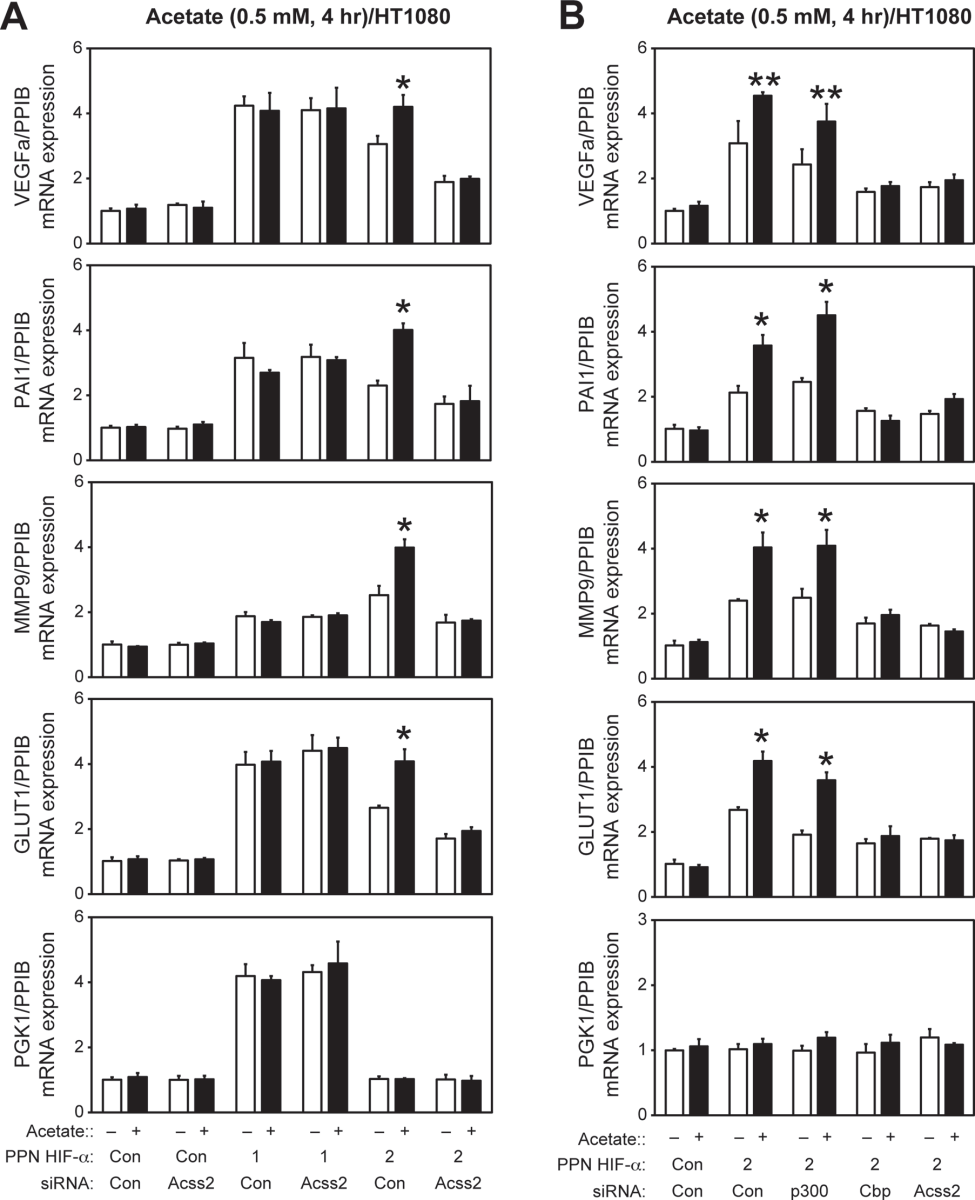
ACSS2/CBP mediate acetate augmentation of HIF-2 signaling. HIF target genes induced in (A) HT1080 cells expressing no ectopic HIF (control), oxygen-insensitive (PPN) HIF-1α, or PPN HIF-2α following control or ACSS2 knockdown, and treated with vehicle or acetate, or in (B) HT1080 cells expressing PPN HIF-2α following control, p300, CBP, or ACSS2 knockdown, and treated with vehicle or acetate. Comparison by one-tailed t-test between vehicle (empty bars) and acetate treatment (filled bars) with significant increases compared to vehicle treatment indicated (single pool of triplicate biological replicates/manipulation; triplicate measurements/pool; mean/SD; *, P<0.05; **, P<0.10).

We also examined the effect of acetate supplementation on HIF signaling mediated by ectopic oxygen-independent HIF-α mutant proteins, which have substitution mutations for the proline and asparagine residues that are hydroxylated by the oxygen-dependent prolyl hydroxylases enzymes (PHD) and asparagine hydroxylase (FIH1), respectively [9]. Acetate augments oxygen-independent HIF-2 signaling, but not oxygen-independent HIF-1 signaling, in an ACSS2-dependent manner; acetate also augments oxygen-independent HIF-2 signaling during glucose deprivation, which does not activate oxygen-independent HIF-1 signaling (Fig. 5A). Acetate augmentation of oxygen-independent HIF-2 signaling occurs in a CBP-dependent manner whereas p300 has no apparent role in this context (Fig. 5B). Hence, acetate-augmented HIF-2 signaling can occur in an oxygen- and glucose-replete environment if oxygen-dependent modifications of HIF-2α are prevented.

### ACSS2 and HIF-2α regulate *in vitro* tumor cell properties

**Fig 6:**
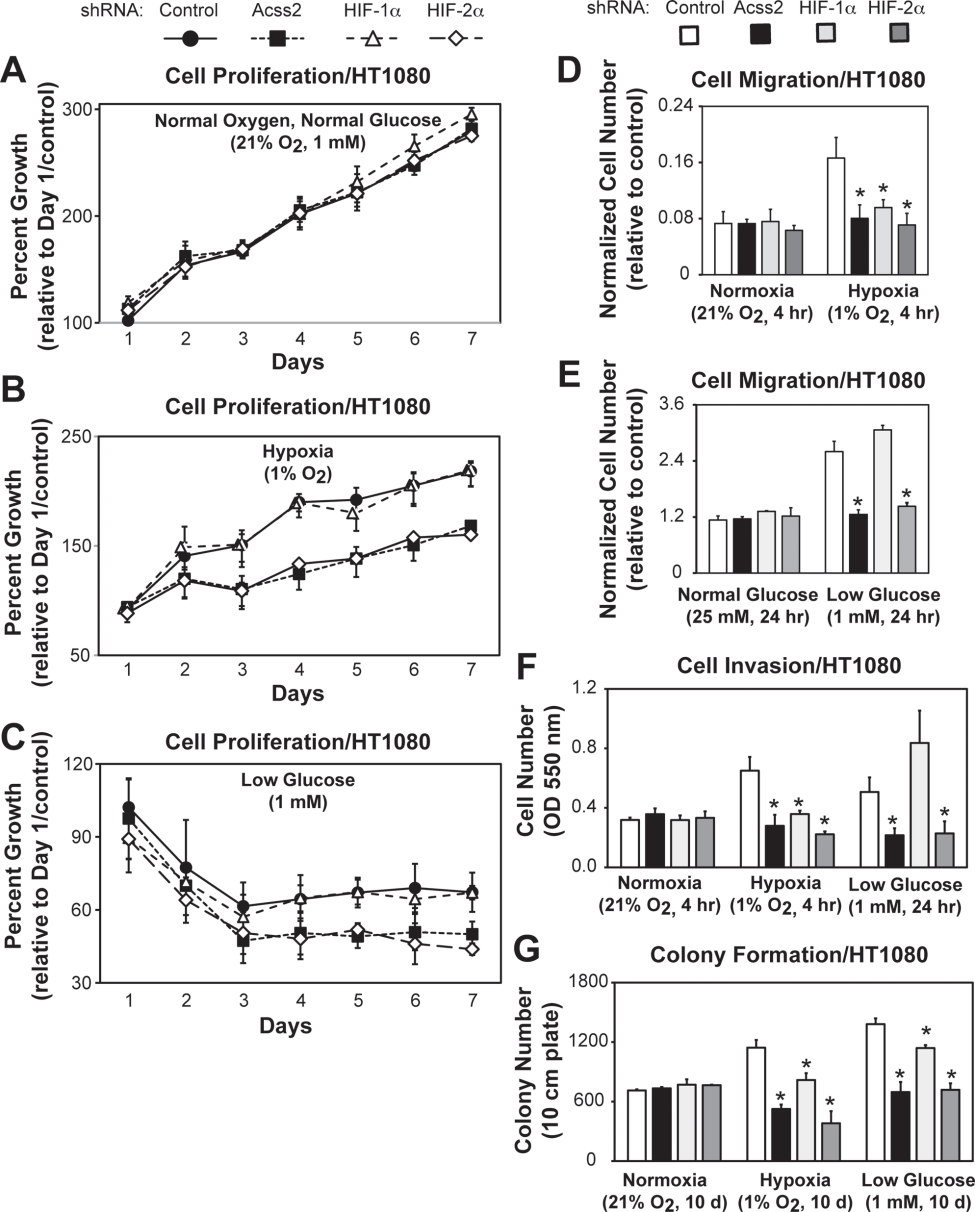
ACSS2 and HIF-2α regulate *in vitro* tumor cell properties. Cell proliferation assessed by cell survival for stably transformed HT1080 cells exposed to either (A) ambient, (B) hypoxic, or (C) low glucose conditions (n = 8/treatment/day) and expressing control (black circles), ACSS2 (black squares), HIF-1α (white triangles), or HIF-2α (white diamonds) shRNA. Cell migration assessed for same control (white bars), ACSS2 (black bars), HIF-1α (light gray bars), or HIF-2α (dark gray bars) knockdown cells exposed to either (D) hypoxic or (E) low glucose conditions (n = 3/treatment). (F) Cell invasion assessed under ambient, hypoxic, or low glucose conditions (n = 3/treatment). (G) Colony formation assessed under ambient, hypoxic, or low glucose conditions (n = 3/treatment). Comparison by one-tailed t-test between control and specified knockdown/treatment with significant reductions compared to control indicated (mean/SD for indicated number of replicates; *, P<0.05).

We asked if reducing ACSS2 or HIF levels affects tumor cell proliferation. ACSS2, HIF-2α, or HIF-1α knockdown has no effect on cell proliferation under oxygen- and glucose-rich conditions (Fig. 6A). However, ACSS2 or HIF-2α, but not HIF-1α, knockdown impairs cell proliferation during hypoxia (Fig. 6B) or low glucose (Fig. 6C) during at least the initial three days. Cell proliferation appears parallel at later time points, although cell growth at these later time-points may be confounded by changes in monolayer cell culture conditions that accompany an increase in cell number [16], including changes that may affect HIF signaling in an isoform-specific manner [17,18,19,20].

We asked if reducing ACSS2 or HIF levels affects other tumor cell properties. Reducing ACSS2 or HIF-2α levels affects cell migration (Fig. 6D-E) and invasion (Fig. 6F), and HIF-1α knockdown also affects these processes during hypoxia. Finally, ACSS2 or HIF-2α knockdown blunts colony formation under hypoxic or low glucose conditions (Fig. 6G). Efficient knockdown of ACSS2, HIF-1α, or HIF-2α is maintained over both short-term (S4A-B Fig.) as well as long-term (S4C-D Fig.) hypoxia or low glucose exposure.

We next asked if ACSS2 and HIF proteins were required for select acetate-responsive cell culture phenotypes. Knockdown of ACSS2 or HIF-2α resulted in blunted colony formation in cells exposed to exogenous acetate (S5 Fig.), but HIF-1α knockdown had no significant effect on this property. ACSS2 has also been implicated in regulating acetate-dependent lipid synthesis in tumor cells. ACSS2, but not HIF-2α, knockdown decreased acetate-dependent lipid synthesis under all conditions (S6 Fig.), whereas HIF-1α knockdown had an intermediate effect under hypoxia exposure, but had no effect under glucose deprivation. Thus, ACSS2 and HIF-2α share similar functional roles in several *in vitro* cell culture models of tumor cell function, as defined by the effect of their absence on these properties, but differ in at least one aspect of intermediary metabolism as defined by their role in acetate-dependent lipid synthesis.

### ACSS2 and HIF-2α regulate *in vivo* tumor cell properties

**Fig 7:**
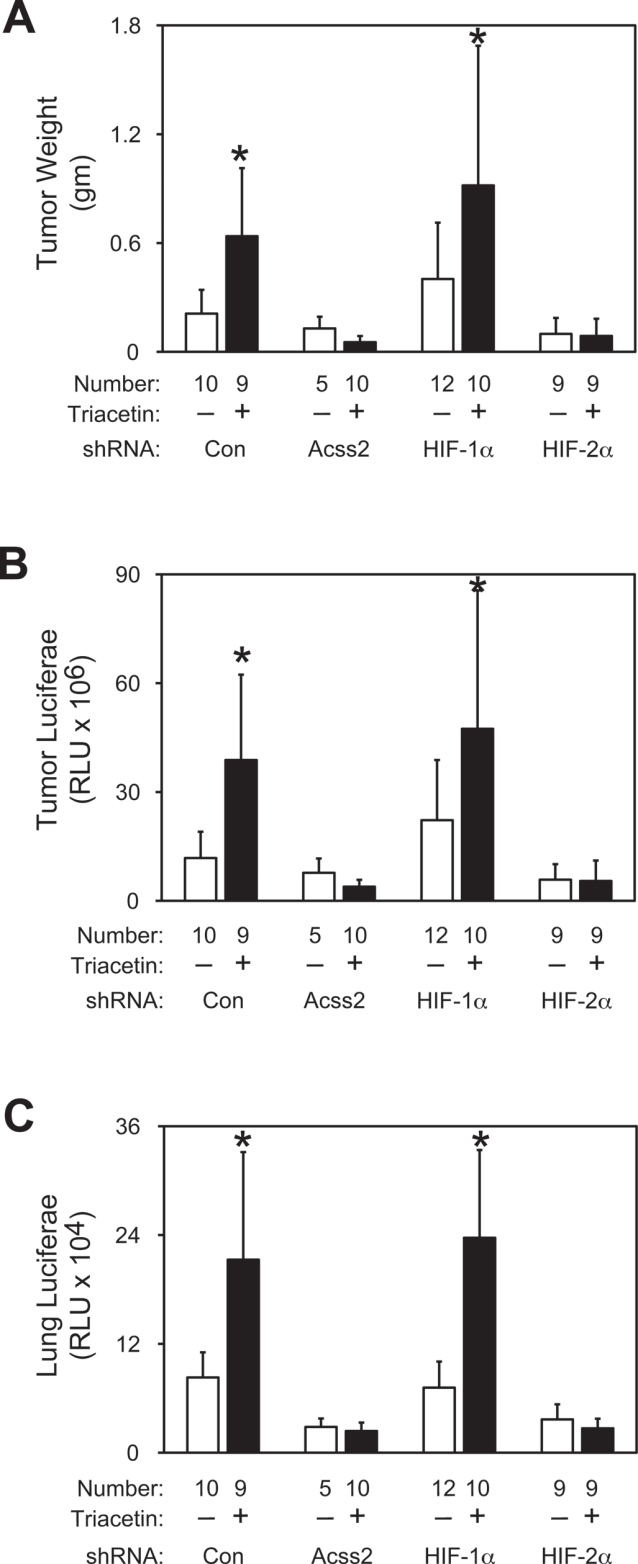
ACSS2 and HIF-2α regulate *in vivo* tumor cell properties. (A) Weights of flank tumors derived from HT1080 cells expressing control (white bars), ACSS2 (black bars), HIF-1α (light gray bars), or HIF-2α (dark gray bars) shRNA downstream of a luciferase cDNA cassette. Tumor-bearing mice received daily vehicle or acetate (triacetin) oral gavage treatments beginning 6 days after tumor cell implantation until euthanization (20 days following tumor cell implantation). (B) Luciferase activity of flank tumor or (C) lung extracts from same mice in (A). For (A) through (C), comparison by one-tailed t-test or z-test between vehicle and acetate treatments with significant increases compared to vehicle treatment indicated (mean/SD for indicated number of mice with tumors; *, P<0.05). Not indicated is the tumor failure mice (no tumor detected at the completion of the experiment), which were as follows: n = 2 control shRNA/vehicle, n = 7 ACSS2 shRNA/vehicle, n = 0 HIF-1α shRNA/vehicle, or n = 3 HIF-2α shRNA/vehicle, n = 0 control shRNA/triacetin, n = 3 ACSS2 shRNA/triacetin, n = 2 HIF-1α shRNA/triacetin, or n = 4 HIF-2α shRNA/triacetin.

We asked whether ACSS2 and HIF signaling affect tumor cell growth and metastases. Mice carrying HT1080 flank tumors expressing control, ACSS2, HIF-1α, or HIF-2α shRNA downstream of a luciferase cDNA were treated daily with vehicle or an acetate triester, triacetin [21]. Tumor burden measured by weight (Fig. 7A) or luciferase activity (Fig. 7B) were substantially reduced for ACSS2 and HIF-2α depleted cells, but not for control or HIF-1α depleted cells. Similarly, lung metastases were also markedly reduced following ACSS2 or HIF-2α knockdown, but not following HIF-1α knockdown (Fig. 7C). Tumor burden and metastases increased with acetate supplementation for control or HIF-1α, but not ACSS2 or HIF-2α, knockdown. Thus, acetate is capable of augmenting tumor growth in an ACSS2 as well as HIF-2, but not HIF-1, dependent manner, consistent with the specific effects of acetate on ACSS2/HIF-2 function at the molecular, biochemical, and cellular level.

## Discussion

**Fig 8:**
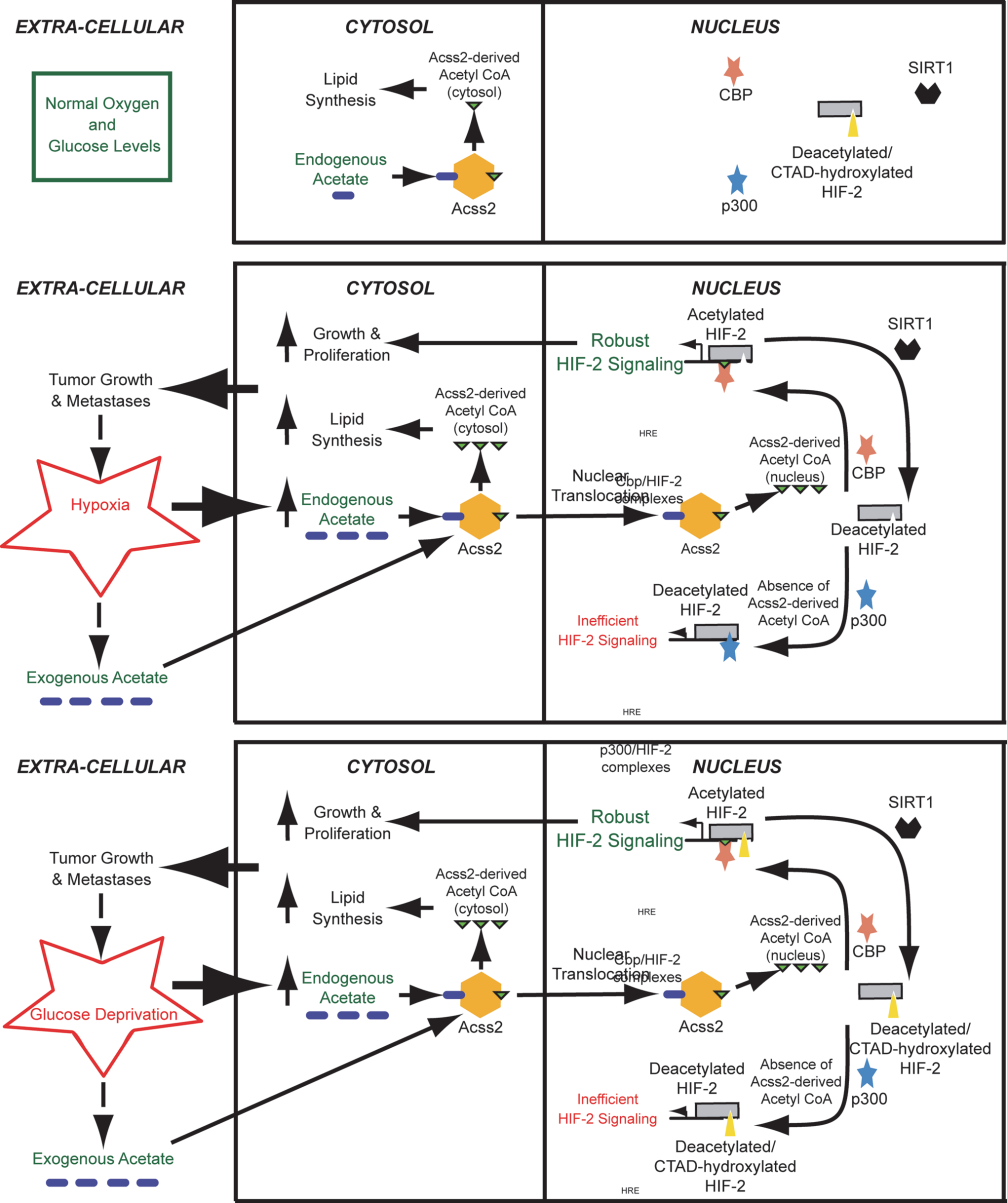
Proposed role of the acetate switch in tumor biology. Endogenous acetate generated in response to hypoxia or glucose deprivation, or exogenous acetate originating from neighboring cells or from gastrointestinal uptake, stimulates ACSS2-dependent acetyl CoA production in the cytosol, but also directs production of an acetyl CoA pool that is localized in the nucleus upon ACSS2 nuclear translocation. In the cytosol, ACSS2 contributes to lipid synthesis, likely for cell growth and proliferation. In the nucleus, the acetyltransferase/coactivator CBP uses this specific ACSS2-derived acetyl CoA pool for HIF-2α acetylation and CBP/HIF-2α complex formation, which augments HIF-2 signaling. CBP is only bound to HIF-2α while it is undergoing the acetylation reaction, which occurs as long as ACSS2-generated acetyl CoA remains available. When acetylation of HIF-2α is complete, CBP is released. SIRT1 then deacetylates HIF-2α, restoring HIF-2α to a CBP substrate. After transformation or as a tumor forms, acetate may be produced constitutively, which further augments tumor growth. In the absence of ACSS2-generated acetyl CoA, HIF-2α complexes with p300 during hypoxia, but not during glucose deprivation. The p300/HIF-2α complex, however, is inefficient at inducing HIF-2 signaling compared to CBP/HIF-2α.

An acetate switch has been described in lower organisms [22] and its presence inferred in higher metazoans [23]. We recently identified an acetate-based biochemical switch in mammalian cells that differentially regulates recruitment of an acetylase/coactivator, CBP, to a stress-responsive transcription factor, HIF-2, during stress induced by hypoxia [10]. Activation of the acetate switch results in production of a specific pool of acetyl CoA by the acetate-dependent acetyl CoA synthetase ACSS2, which plays a specialized and context-dependent signaling role in HIF-2 regulated erythropoietin gene expression and in stress-induced erythropoiesis during both acute and chronic anemia. In this study, we find that the acetate switch is relevant in another pathophysiological condition with activated HIF-2 signaling, cancer, and in a stress state found in solid tumors besides hypoxia, glucose deprivation (Fig. 8).

ACSS2 was initially proposed as a cytosolic acetyl CoA source for lipid biosynthesis [15]. In mammalian cells, cytosolic acetyl CoA generated by ATP citrate lyase, the major source of acetyl CoA during normal growth conditions, regulates growth factor-induced histone acetylation [24]. Under hypoxia or with mitochondrial dysfunction, citrate production in transformed cells is maintained despite impairment in the forward flow of the TCA cycle through reductive carboxylation [25,26,27,28]. Even so, our findings indicate that ATP citrate lyase does not participate in HIF signaling during hypoxia or in HIF-2 signaling for HT1080 cells during low glucose conditions, which does not induce HIF-1 signaling in HT1080 and other cells [13].

Acetate release from cancer cells increases during prolonged hypoxia and depletion of ACSS2 affects lipid composition [29,30]. We demonstrate herein that acetate levels rise acutely following stress exposure and induce signaling effects within minutes as evident by acetylation of HIF-2α. Thus, the initial function of acetyl CoA generated by ACSS2 may be to activate select stress-signaling pathways such as HIF-2, which precedes any significant use of acetate-derived acetyl CoA in intermediary metabolic processes. A decrease in acetate below threshold levels reduces acetyl CoA production by ACSS2. However, stress-induced reductions in co-factors that are required for ACSS2-dependent acetyl CoA generation, such as ATP, may confer additional links of the acetate switch with energy and nutrient sensing.

Acetate and ATP levels may change with hypoxia or glucose deprivation, but the time-course and extent of ATP reduction in fibrosarcoma-derived HT1080 cells likely differs based upon our *in vitro* biochemical and cell culture data. During early hypoxia, acetate increases and ATP levels are sufficient to allow ACSS2 function, which promotes CBP/HIF-2α complex formation. As hypoxia progresses, acetate, and likely ATP, levels decrease, which inhibits ACSS2 function and thus CBP/HIF-2α complex formation. In the absence of ACSS2, p300/HIF-2α complexes form, but are evidently inadequate for efficient activation of HIF-2 target genes. During glucose deprivation, acetate induction occurs late, but ATP levels likely remain adequate for ACSS2 function, so the acetate switch is activated. Similar results of decreased ATP levels observed after hypoxia, but not low glucose, exposure have been made with immortalized mouse embryonic fibroblasts [31].

Our data indicate that ACSS2/HIF-2 signaling is active in transformed cells exposed to hypoxia and glucose deprivation, environmental stresses experienced by cells growing in solid tumors. Similar to results from earlier studies [30], we found that acetate-dependent lipid synthesis in HT1080 cells exposed to chronic hypoxia is altered by ACSS2 knockdown. We also noted that acetate-dependent lipid synthesis via ACSS2 occurs during glucose deprivation as well as under ambient conditions. Although gain [32,33] or loss of [34] function in HIF-2 signaling may influence lipid homeostasis in liver, our results suggest that impaired HIF-2 signaling does not affect acute changes in acetate-dependent lipid synthesis in HT1080 cells under either ambient, hypoxia, or low glucose conditions.

## Conclusions

The ACSS2/CBP/SIRT1/HIF-2 axis links substrate-dependent co-activator recruitment to stress signaling induced by hypoxia, glucose deprivation, and likely other environmental stresses. Stress signaling in this pathway is conferred by cyclical acetylation/deacetylation modifications of HIF-2, which in turn are regulated by availability of cellular metabolites used as substrates or cofactors in the ACSS2-regulated and CBP-mediated acetylation of HIF-2. Our finding that exogenous acetate promotes cancer growth and metastases in an animal model via ACSS2 and HIF-2 identifies this pathway as one that potentially may be influenced by environmental cues.

Defining the inner workings of the mammalian acetate switch will illuminate how intermediary metabolism and stress recognition are exquisitely linked to the prosurvival cellular response regulated by ACSS2/HIF-2 in normal and abnormal mammalian physiology. Intentional manipulation of the ACSS2/HIF-2 signaling pathway may have desirable effects in human disease, although whether this occurs through stimulation or inhibition will depend upon the specific role that ACSS2/HIF-2 signaling plays in the physiological or pathophysiological process of interest.

## Materials and Methods

### Cell culture and transfections

We maintained HT1080 cells (Cat. No. CCL-121, ATCC, Manassas, VA) as previously described [14] in complete DMEM media (Cat. No. SH30022, HyClone, Logan, UT), 10% fetal bovine serum (FBS; Cat. No. F4135, Sigma, St. Louis, MO) with penicillin (100 U mL^-1^)/streptomycin (100 μg mL^-1^) (Cat. No. 15140–148, Gibco BRL, Carlsbad, CA) in a 5% CO_2_, 95% air incubator. We assayed cells periodically for mycoplasma. For hypoxia exposure, cells were cultured with standard glucose media (25 mM glucose) under hypoxic conditions (1% oxygen, 5% carbon dioxide, 94% nitrogen) in a hypoxia workstation (Coy Laboratories). For glucose deprivation, cells were incubated with low glucose media (1 mM glucose) under normoxic conditions (21% oxygen, 5% carbon dioxide, 74% nitrogen) in a standard tissue culture incubator. For short chain fatty acid (SCFA) addition, we added the indicated sterile stocks of SCFA (acetate, proprionate, butyrate) to complete medium (final concentration 0.5 mM) and harvested cells after the indicated time under normal oxygen conditions. We prepared extracts from hypoxia-exposed cells within a hypoxia workstation (Coy Laboratory Products, Inc., Grass Lake, MI). We prepared extracts from cells maintained under low glucose conditions or after SCFA treatment under normal oxygen conditions. For sequential transfection of HT1080 cells with siRNA and plasmid DNA, we replated cells after siRNA transfection and transfected plasmid DNA using Lipofectamine 2000 (Cat. No.15338–100, Invitrogen, Carlsbad, CA) as previously described [14]. For cellular assays (proliferation, migration, invasion, colony formation), we generated stable HT1080 cell lines by lentiviral transduction and antibiotic selection that express control, ACSS2, HIF-1α, or HIF-2α miR30 shRNA downstream of a DsRed cDNA. For mouse studies (flank tumor), we generated stable HT1080 cell lines expressing these same shRNA downstream of a firefly luciferase cDNA.

### Lentivirus stable transduction

Lentiviruses were generated by co-transfection of shuttle vectors with packaging plasmids psPAX2 and pMD2G. The day before transduction, HT1080 cells were trypsinized and 2 x 10^5^ cells per well plated in 1 mL complete culture medium in a 6-well plate for overnight incubation at 37°C. On the day of transduction, media was removed and replaced with 1 ml of complete medium with 10 μg/ml polybrene (Cat. No. 107689, Sigma). Lentiviral particles were thawed to room temperature, mixed gently, and added to the HT1080 cells (MOI = 30). After gently swirling to mix, cells were incubated overnight. After 12 hr, culture medium was replaced with 2 ml of complete medium containing 10 μg/ml blasticidin S (Cat. No. ant-bl, Invivogen, San Diego, CA), which was replaced every 2 days until one week after all control cells had died. Positive cells were maintained in 1 μg/ml blasticidin S for 2 weeks, and then were frozen down until use. For experiments, cells were thawed and allowed to grow for three passages before use.

### HIF-2α expression plasmids

Wild-type (WT) and oxygen-independent (PPN) HIF-2α expression plasmids were made as previously described [9]. All constructs contain a carboxy terminal hemagglutinin A (HA) epitope tag. Constructs used in co-immunoprecipitations and pulldowns also have an S-protein (SP) tag at the amino terminus to allow for facile purification [35].

### siRNA knockdown

For siRNA knockdown, we transfected HT1080 cells in a 6-well plate with siRNA (ThermoFisher Scientific, Lafayette, CO) directed against control (Cat. No. D-001810-10-20), ACLY (Cat. No. L-004915-00-0005), ACSS1 (Cat. No. L-008549-01-0005, ACSS2 (Cat. No. L-010396-00-0005), p300 (Cat. No. L-003486-00-0005), CBP (Cat. No. L-003477-00-0005), SIRT1 (Cat. No. L-003540-00-0005), HIF-1α (Cat. No. L-004018-00-0005), EPAS1/HIF-2α (Cat. No. L-004814-00-0005), or FIH1 (Cat. No. L-004073-00-0005) using DharmaFECT1 (Cat. No. T-2001-03, Thermo Fisher Scientific, Lafayette, CO) as previously described.

### shRNA knockdown

For lentiviral transduction knockdown studies, we generated lentiviral (LTV) expression shuttle vectors (pLenti6/V5-GW/lacZ, Invitrogen) based upon similar constructs that we have previously described [10]. We replaced the lacZ-encoding cDNA with a DsRed (for cell studies) or firefly luciferase (for mouse flank tumor studies) cDNA followed by a polylinker containing a concatamer of four different shRNAs that target the gene of interest (ACSS2, HIF-1α, or HIF-2α).

### HIF-1α and HIF-2α protein time-course experiments

To define endogenous protein expression patterns, wild-type HT1080 cells in twenty-four 10 cm plates were plated on Day-1. On Day 0, cells were placed under hypoxia in a hypoxia workstation or under low glucose conditions in a standard tissue-culture incubator for 0, 2, 4, 8, 16, or 24 hr until harvest. At the indicated time period, cells were rinsed with PBS once and then were scraped with1 ml CytoBuster protein extraction reagent (Cat. No. 71009, Novagen, Gibbstown, NJ) plus 1x Protein Inhibitor Cocktail (Cat. No. P8340; Sigma). The samples were stored in a liquid nitrogen air-phase overnight and then used the following day to generate whole cell extracts by thawing samples on ice, vortexing at high speed for 15 sec, and pelleting cellular debris for 15 min at 20,000g in a 4°C coldroom. The cleared supernatant was transferred to a new tube and a portion was used to determine protein concentrations (BCA Assay; Cat. No. 23225, Thermoscientific). For hypoxia samples, reagents were allowed to equilibrate in the hypoxia chamber for 2 hr prior to use. To detect endogenous HIF-1α and HIF-2α, whole cell extract (10 ug) was electrophoresed on 10% SDS-PAGE gels along with a protein ladder and transferred to a 0.45 uM PVDF membrane (Immobilon-P; Cat. No. IPVH00010, Millipore) for 3 hr at 60 V in a 4°C coldroom. The membrane was cut to allow for determination of HIF-1α or HIF-2α as well as alpha-tubulin from the same experiment. The membrane was blocked with TBST with 5% non-fat drymilk at room temperature for 1 hr, and then incubated with primary antibody in blocking solution overnight (∼16 hr) in a 4°C coldroom with gentle shaking. Membranes were then rinsed five times, ∼5 min/rinse with TBST (TBS with 1% Tween-20). Next, membranes were incubated with the appropriate secondary antibody for 1 hr, room temperature. Membranes were rinsed again five times, ∼5 min/rinse with TBST and then incubated with the indicated detection reagent, followed by visualization with plain film exposure. The primary antibodies used were anti-human HIF-1α mouse monoclonal (1:1,000; Cat. No. 610959, BD Biosciences, San Jose, CA), anti-human HIF-2α mouse monoclonal (1:1,000 dilution; Cat. No. NB100-132, Novus Biologicals, Littleton, CO) or anti-human HIF-2α rabbit polyclonal (1:1,000 dilution; Cat. No. NB100-122, Novus Biologicals), anti-HA mouse monoclonal antibody (1:1,000 dilution; Cat. No. H9658, Sigma), or alpha-tubulin (1:3,000 dilution; Cat. No. T9026, Sigma) antibodies. The secondary antibodies used were horseradish peroxidases-linked goat anti-mouse (1:3,000 for HIF-1, 1:2,500 for HIF-2; Cat. No. 7076S, Cell Signaling Technologies) or goat anti-rabbit (1:2,500 for HIF-2, 1:25,000 for alpha-tubulin; Cat. No. 7074S, Cell Signaling Technologies, Danvers, MA) antibodies. The detection reagents used include Super Signal West Dura Extended Duration (for HIF-2 mouse monoclonal antibody; Cat. No. 34076, Thermoscientific) or Clarity (remainder immunoblots; Cat. No. 170–5061, BioRad).

For ectopic HIF-2α time-course experiments, a cDNA encoding amino-terminal S-peptide (SP) epitope tagged and carboxy-terminal hemagglutinin A (HA) epitope tagged WT HIF-2α (SP:WT HIF-2α:HA) was cloned into a modified lentiviral expression shuttle vector (pLenti6/V5-GW/lacZ, Invitrogen) in place of the lacZ cDNA and this plasmid was used to generate lentivirus (LTV) stocks as described above. To express SP:WT HIF-2α:HA in HT1080 cells, 6 cm plates of HT1080 cells were infected with excess LTV and allowed to express for 48 hr before beginning the hypoxia or low glucose exposure experiments.

### HIF-2α acetylation experiments

For ectopic HIF-2α acetylation experiments, amino-terminal S-peptide (SP) epitope tagged and carboxy-terminal hemagglutinin A (HA) epitope tagged WT HIF-2α (SP:WT HIF-2α:HA) expressed in HT1080 cells was purified using SP-agarose and subjected to immunoblot analyses as previously described [9]. For endogenous HIF-2α acetylation experiments, HIF-2α was immunoprecipitated and acetylated as well as total HIF-2αα was detected by immunoblotting with a mouse monoclonal antibody as previously described [10]. In all cases, SIRT1 inhibitor treatment with sirtinol and nicotinamide (NAM) was initiated 6 hr prior to harvest of samples.

### Histone H3 purification and acetylation detection

HT1080 cells grown to 80% confluence and maintained under standard growth conditions were treated with media supplemented with sodium acetate, sodium propionate or sodium butyrate (0.5 mM final concentration) for 4 hr. Histone H3 protein were purified with a histone purification kit (Cat. No. 40026, Active Motif, Carlsbad, CA) according to the manufacturer’s instructions. Briefly, cells were lysed in extraction buffer at 4°C for 1 hr on a rotating platform. Cleared lysates were neutralized by addition of five volumes neutralizing buffer, and then loaded on pre-equilibrated columns packed with purification resin. Columns were extensively washed with histone wash buffer, and histones were eluted in 0.5 ml fractions using the supplied elution buffer. Purified histone preparations were quantified by measuring absorbance at 230 nm, and then detected by immunoblot analysis using histone H3 antibody (Cat. No. 61278, Active Motif), histone H3-K4ac antibody (Cat. No. 39382, Active Motif), or histone H3-K9ac antibody (Cat. No. 39138, Active Motif).

### Endogenous protein immunoprecipitation experiments

Immunoprecipitation of endogenous proteins was accomplished with a Universal Co-IP kit (Cat. No. 54002, Active Motif) according to the manufacturer’s protocol. HT1080 nuclear extracts were first incubated with protein A agarose beads. Cleared supernatants were incubated with EPAS1 (HIF-2α) antibody (Cat. No. NB100-132, Novus Biologicals) or normal mouse IgG (Cat. No. sc-2025, Santa Cruz Biotechnology, Santa Cruz, CA) 2 hr before addition of protein A agarose beads. After binding, beads were pelleted by centrifugation and washed. After washing, immunoprecipitated materials were eluted and immunoblotted with anti-human p300 (1:500 dilution; Cat. No. sc-584, Santa Cruz Biotechnology), anti-human CBP (1:500 dilution; Cat. No. 4772, Cell Signaling Technology), or anti-human HIF-2α mouse monoclonal (1:1,000 dilution; Cat. No. NB100-132, Novus Biologicals) antibodies.

### *In vitro* immunoprecipitation experiments

For the siRNA knockdown experiments, we first transfected HT1080 cells with siRNA, waited 48 hr, exposed cells to the indicated condition, and prepared nuclear extracts as described [9]. We added acetyl CoA (Cat. No. A2056, Sigma; final concentration 50 μM), ATP (Cat. No. FLAAS-1VL, Sigma; final concentration 100 μM), or acetate (Cat. No. A2056, Sigma; final concentration 50 μM) to 50 μl whole cell extract prepared using a Universal CoIP Kit (Cat. No. 54002, Active Motif) and incubated the extract at 30°C for 30 min. We then immunoprecipitated endogenous HIF-2α and performed immunoblotting for CBP or p300 as described. For hypoxia samples, all extract preparations, additions, and immunoprecipitations were performed within the hypoxia workstation.

### Cell fractionation and immunoblotting

To prepare extracts, we used CytoBuster protein extraction reagent (Cat. No. 71009, Novagen, Gibbstown, NJ) or NE-PER nuclear and cytoplasmic extraction reagents (Cat. No. 78833, Pierce, Rockford, IL) as previously described [9]. Proteins were analyzed by immunoblotting with anti-human p300 (1:500 dilution; Cat. No. sc-584, Santa Cruz Biotechnology), anti-human CBP (1:500 dilution; Cat. No. 4772, Cell Signaling Technology), anti-human ACLY (1:1,000 dilution; Cat. No. 4332, Cell Signaling Technology), anti-human ACSS1 (1:1,000 dilution; Cat. No. SAB1400745, Sigma), anti-human ACSS2 (1:500 dilution; Cat. No. ab66038, Abcam, Cambridge, MA), anti-human HIF-2α (1:1,000 dilution; Cat. No. NB100-132, Novus Biologicals), anti-TATA-binding protein (TBP) (1:1,000 dilution; Cat. No. sc-204, Santa Cruz Biotechnology), anti-α-tubulin (1:10,000 dilution; Cat. No. T9026, Sigma), or anti-HA (1:5,000 dilution; Cat. No. H9658, Sigma) antibodies as indicated.

### Acetate determinations

We transferred HT1080 cells grown to 80% confluence under standard growth conditions to an incubator within the hypoxia workstation after changing the standard media or maintained the cells within the standard incubator after changing to low glucose media until the indicated time-point. At the time of harvest, we aspirated off media, quickly added 1 ml ice-cold 0.1 N HCl and scraped cells with a spatula. After transferring the lysate to an ice-cold microfuge tube, cellular debris was pelleted at 14,000 rpm, 4°C, 10 min. We transferred 0.9 ml supernatant to a new, ice-cold microfuge tube, and then adjusted the pH to 6.5–7.0 with 5 N NaOH. Extracts were stored at -80°C until assay. Acetate concentrations were determined with the Megazyme Acetic Acid Rapid Kit (Cat. No. K-ACETRM, Megazyme, Ireland) according to the manufacturer’s protocol as previously described [10].

### Real-time PCR analyses

The expression of endogenous *VEGFa*, *PAI1*, *MMP9*, *GLUT1*, *PGK1*, and *PPIB* in HT1080 cells were determined by reverse transcription of total RNA followed by semi-quantitative real-time PCR analysis on an Applied Biosystems ABI Prism 7000 thermocycler using Power SYBR Green Master Mix following the manufacturer’s protocol as previously described [9]. We determined the relative levels of gene expression from a single pooled sample made from three individually transfected wells (biological replicates) of HT1080 cells in a 12-well plate for each condition. The results of triplicate experiments, with each sample measured as triplicates, were expressed as 2 ^-(gene-of-interest number of cycles- cyclophilin number of cycles)^.

### Colony Formation

Five hundred HT1080 cells were seeded in triplicate 10 cm plates and allowed to attach for 24 hr. After 24 hr, cells were treated with complete media at 1% oxygen or media containing 1 mM glucose or 5 mM acetate at 21% oxygen for 10 days. Media was not changed throughout the experiment. Colonies were then stained with 1% crystal violet dissolved in ethanol/PBS (15%/85%). Cells were imaged and colony number determined using ImageJ software.

### Cell proliferation assays

1 x 10^3^ HT1080 cells/well were seeded in a 96-well plate with each cell line in replicate sets of eight. After 24 h, cells were exposed for 1 week to 1% oxygen, or exposed to 21% oxygen with either standard glucose media (25 mM glucose), or low glucose media (1 mM glucose). Media was changed every 48 hr with comparable media. Cell proliferation was detected every day with the CellTiter 96 AQueous Non-Radioactive Cell Proliferation Kit (Cat. No. G5421, Promega, Madison, WI) according to the manufacturer’s protocol. The absorbance was recorded at 490 nm with a microplate reader.

### Cell migration and cell invasion assays

For cell migration assays, HT1080 cells were serum-starved in 0.5% FBS/DMEM media overnight. After 12 hr, 1.5 x 10^5^ HT1080 cells in serum-free media were transferred into a transwell insert. For cells maintained under normal conditions, cells were incubated with complete media and exposed to 21% oxygen for 4 h. For cells maintained under hypoxic conditions, cells were incubated with complete media and exposed to 1% oxygen for 4 hr. For cells exposed to low glucose conditions, cell were incubated with low glucose (1 mM) media at 21% oxygen for 24 hr and compared to control cells maintained under standard glucose (25 mM) conditions for 24 hr. Cell migration was detected from triplicates for each treatment after crystal violet staining. The absorbance was recorded at 560 nm with a microplate reader.

For cell invasion assays, we used a commercially available kit containing wells pre-filled with Matrigel (CytoSelect 24-Well Cell Invasion Assay Kit; Cat. No. CBA-110, Cell Biolabs, San Diego, CA). HT1080 cells were serum-starved in 0.5% FBS/DMEM media overnight. After 12 hr, 1.5 x 10^5^ HT1080 cells in serum-free media were transferred into the transwell insert as above after pre-incubating the transwell insert for 1 hr with serum-free media at room temperature. Cell migration was determined from triplicates for each treatment according to the manufacturer’s protocol.

### ^14^C-Acetate lipid synthesis determinations

HT1080 cells were plated as triplicates for each experimental sample in 6 cm or 6-well plates at 60% confluence and allowed to attach overnight under standard growth conditions. The next morning, cells were housed under ambient (21% oxygen, 10 mM glucose), hypoxic (1% oxygen), or low glucose (1 mM glucose) conditions. Twenty-four hours later, media containing ^14^C-acetate [Perkin Elmer, Catalog Number NEC553050UC, Acetic Acid, Sodium Salt, (1,2–^14^C), Santa Clara, CA] was added and the cells grown under the same conditions for an additional twenty-four hours. At the time of harvest, we aspirated off media, rinsed cells twice with 1x PBS, and added Triton-X 100 (0.5% in ddH_2_O) to solubilize cells. We removed an aliquot for total protein determinations. After transferring the lysis to a microfuge tube, the cellular debris was pelleted at 14,000 rpm, 4°C, 10 min. We performed lipid extraction with sequential addition of methanol, chloroform, and water with vortexing and centrifugation after each step to resolve the aqueous and organic phases. After the last centrifugation, the organic phase was transferred to a new tube and evaporated to dryness under air in a hood. Extracts were resuspended in 100 μl chloroform and counted with Ecolume liquid scintillation cocktail (MP Biomedicals, Cat. No. 0188247001, Santa Ana, CA). Counts were normalized to protein concentration for each sample.

### *In vivo* nude mice flank tumor experiments

All animal experiments were approved by the University of Texas Southwestern Medical Center Institutional Animal Care and Use Committee. Female mice were used exclusively for HT1080 tumor cell implantation. For flank tumor studies, mice were injected subcutaneously on the left dorsal flanks with 5×10^6^ luciferase-expressing stably transformed HT1080 cells (control, ACSS2, HIF-1α, or HIF-2α knockdown) grown using 10% FBS were resuspended in 0.5 ml DMEM. Tumor sizes were measured using calipers every other day beginning on the fourth day after cell injections. Beginning six days after injection of cells, mice were given an acetate ester, triacetin (5.8 gm/kg body weight in PBS; Cat. No. W200700, Sigma-Aldrich Chemicals, Saint Louis, MO), or vehicle (PBS, 0.01 mL/g body weight) by oral gavage once per day. Mice were harvested when the mean tumor volume of any group approached 2 cm^3^. All experiments were terminated at this time-point.

### *Ex vivo* nude mice flank tumor experiments

At the completion of the tumor study, mice were sacrificed and lung as well as tumors removed for biochemical luciferase activity determination as described (Promega, Madison, WI). Individual tissues were weighed and homogenized using a PowerGen 700D homogenizer (ThermoFisher Scientific, Waltham, MA) in lysis reagent (25 mM Tris-phosphate pH 7.8, 2 mM DTT, 2mM 1,2 diaminocyclohexane-N,N,N,N-tetra-acetic acid, 10% glycerol, 1% NP-40) containing soybean trypsin inhibitor (0.2 mg/ml) and bovine serum albumin (0.2 mg/ml). Samples (2 μl tumor or 20 μl lung lysates) were diluted in 100 μl lysis reagent containing 2.5 mM MgCl_2_. Immediately prior to measurement, 50 μl luciferin reagent (20 mM tricine, 1 mM (MgCO_3_)_4_Mg(OH)_2_^.^5H_2_O, 2.67 mM MgSO_4_, 0.1 mM EDTA, 33 mM DTT, 0.27 mM coenzyme Q, 0.47 mM luciferin, 0.53 mM ATP, pH 7.8) was added and measurement performed for 10 sec in a single-tube luminometer (Sirius, Berthold Detection Systems, Pforzheim, Germany).

### Statistical analyses

Where indicated, data were presented as mean with standard deviation (SD) or standard error of the mean (SEM). We compared results obtained from the indicated experimental groups by unpaired Student’s t-Test with Welch’s correction for groups of equal sample size or by z-Test for groups of unequal sample size. One-tailed or two-tailed analyses were performed as indicated. We assumed equal variances for experimental groups. We used one-way ANOVA analyses for multiple comparisons with Dunnett’s multiple comparison posthoc test. The statistical analyses were performed using StatPlus and Prism. All P values less than or equal to 0.05 (*) or 0.10 (**) are reported for the stated comparisons.

### Ethics statement

All animal experiments were approved by the University of Texas Southwestern Medical Center Institutional Animal Care and Use Committee.

## Supporting Information

S1 Fig Conditioned media induces HIF-2α acetylation. (A) Endogenous HIF-2α acetylation detected by immunoblotting (IB) after immunoprecipitation (IP) and following early (4 hr) or late (16 hr) hypoxia exposure with pharmacological inhibition of Sirt1 by sirtinol and nicotinamide (NAM). (B) Same as (A) except after early (2 hr) and late (24 hr) low glucose exposure.
(TIF)

S2 Fig CBP/HIF-2α interactions change during stress. (A) Endogenous CBP/HIF-2α or p300/HIF-2α complexes detected by immunoblotting (IB) after early (4 hr) or late (16 hr) hypoxia exposure following SIRT1, CBP, or combined SIRT1/CBP knockdown. (B) Same as (A) except after early (2 hr) and late (24 hr) low glucose exposure. (C) Same as (A) except after (4 hr) acetate exposure. (D) Endogenous CBP/HIF-2α or p300/HIF-2α complexes after early (4 hr) hypoxia exposure following FIH1, ACSS2, or combined FIH1/ACSS2 knockdown. (E) Same as (D) except after late (24 hr) low glucose exposure. (F) Same as (D) except after (4 hr) acetate exposure.
(TIF)

S3 Fig ACSS2-generated acetyl CoA controls CBP/HIF-2α interactions during stress. (A) Endogenous CBP/HIF-2α or p300/HIF-2α complexes induced by acetyl CoA addition to HT1080 whole cell extracts prepared following ACSS2 knockdown and 4 hr hypoxia exposure. (B) Endogenous CBP/HIF-2α or p300/HIF-2α complexes induced by acetyl CoA, acetate, ATP, or acetate plus ATP addition to HT1080 whole cell extracts prepared after 16 hr hypoxia exposure. (C) Endogenous CBP/HIF-2α or p300/HIF-2α complexes induced by acetyl CoA, acetate, ATP, or acetate plus ATP addition to HT1080 whole cell extracts prepared after 2 hr low glucose exposure. (D) Endogenous CBP/HIF-2α or p300/HIF-2α complexes induced by acetyl CoA addition to HT1080 whole cell extracts prepared following ACSS2 knockdown and 24 hr low glucose exposure.
(TIF)

S4 Fig ACSS2, HIF-1α or HIF-2α knockdown results in reduced target protein levels. ACSS2, HIF-1α, or HIF-2α protein levels in the indicated stable knockdown cell line following (A) short-term hypoxia (4 hr), (B) short-term glucose deprivation (24 hr), (C) long-term hypoxia (7 day) or (D) long-term glucose deprivation (7 day) stress conditions. HIF-1α protein levels are markedly induced during hypoxia, but are virtually undetectable under basal or low glucose conditions. ACSS2 and HIF-2α protein levels are detectable under all conditions and increase modestly during hypoxia, but not during low glucose condtions. Knockdown of ACSS2, HIF-1α (where detected), or HIF-2α protein in the respective cell line was efficient regardless of either the nature or duration of stress exposure. All experiments performed with HT1080 whole cell extracts.
(TIF)

S5 Fig Acetate induces colony formation via ACSS2 and HIF-2α. Colony formation for stably transformed HT1080 cells expressing control (white bars), ACSS2 (black bars), HIF-1α (light gray bars), or HIF-2α (dark gray bars) shRNA following (10 d) acetate exposure. Comparison by one-tailed t-test between control and specified knockdown/treatment with significant reductions compared to control indicated (n = 3/treatment; mean/SD; *, P<0.05).
(TIF)

S6 Fig ACSS2 controls acetate-dependent lipid synthesis. Lipid synthesis as measured by ^14^C-acetate incorporation in stably transformed HT1080 cells expressing control (white bars), ACSS2 (black bars), HIF-1α (light gray bars), or HIF-2α (dark gray bars) shRNA following (24 hr) normoxia, hypoxia, or low glucose exposure. Comparison by one-tailed t-test between control knockdown/treatment and specified knockdown/treatment with significant reductions compared to control indicated (n = 3/treatment; mean/SD; *, P<0.05).
(TIF)
