# Correction: The Acetate/ACSS2 Switch Regulates HIF-2 Stress Signaling in the Tumor Cell Microenvironment

**DOI:** 10.1371/journal.pone.0123612

**Published:** 2015-03-30

**Authors:** The PLOS ONE Staff

There are a number of errors in Fig. 8, “Proposed role of the acetate switch in tumor biology.” Please see the corrected Fig. 8 here:

**Fig 8:**
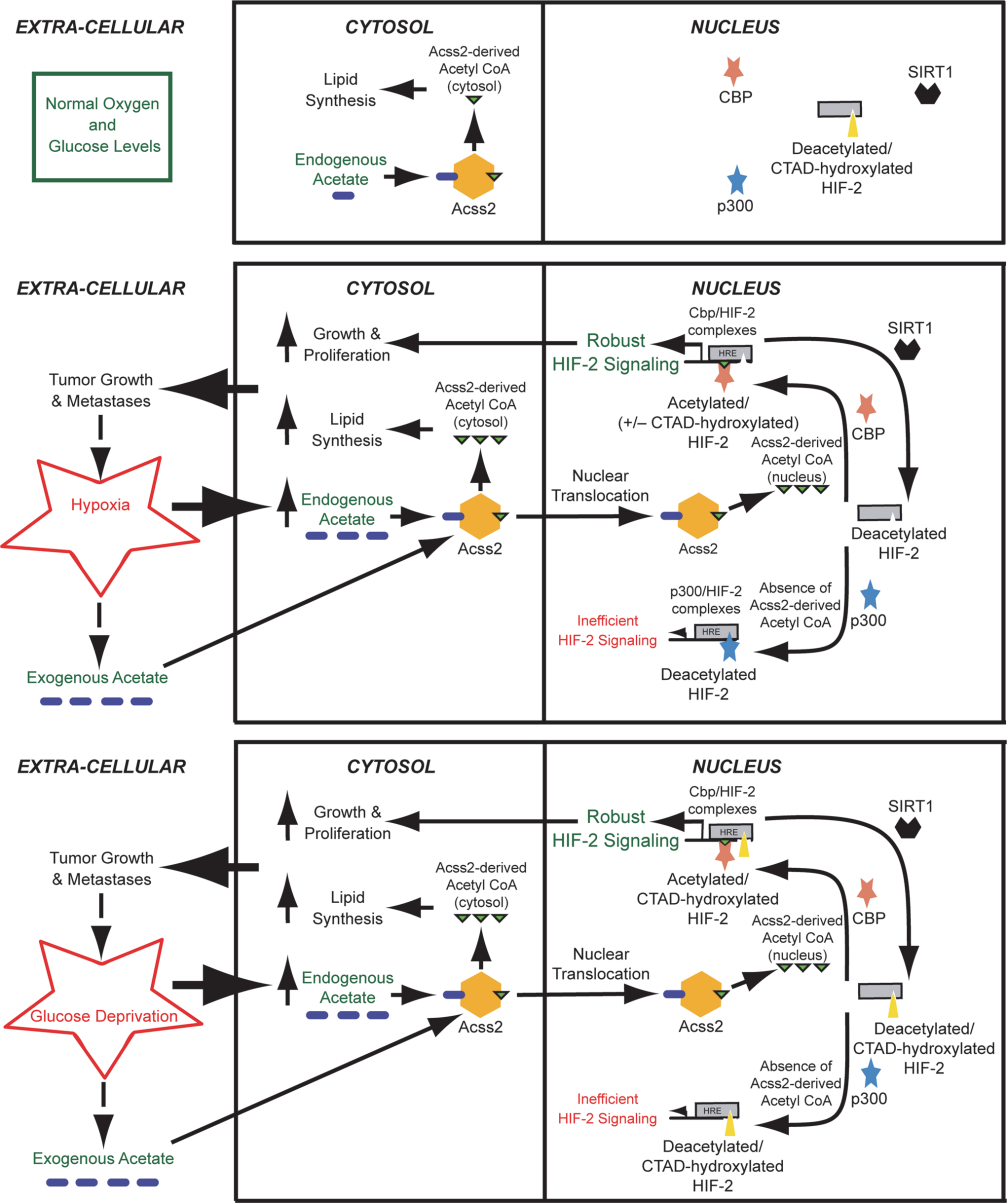
Proposed role of the acetate switch in tumor biology. Endogenous acetate generated in response to hypoxia or glucose deprivation, or exogenous acetate originating from neighboring cells or from gastrointestinal uptake, stimulates ACSS2-dependent acetyl CoA production in the cytosol, but also directs production of an acetyl CoA pool that is localized in the nucleus upon ACSS2 nuclear translocation. In the cytosol, ACSS2 contributes to lipid synthesis, likely for cell growth and proliferation. In the nucleus, the acetyltransferase/coactivator CBP uses this specific ACSS2-derived acetyl CoA pool for HIF-2α acetylation and CBP/HIF-2α complex formation, which augments HIF-2 signaling. CBP is only bound to HIF-2α while it is undergoing the acetylation reaction, which occurs as long as ACSS2-generated acetyl CoA remains available. When acetylation of HIF-2α is complete, CBP is released. SIRT1 then deacetylates HIF-2α, restoring HIF-2α to a CBP substrate. After transformation or as a tumor forms, acetate may be produced constitutively, which further augments tumor growth. In the absence of ACSS2-generated acetyl CoA, HIF-2α complexes with p300 during hypoxia, but not during glucose deprivation. The p300/HIF-2α complex, however, is inefficient at inducing HIF-2 signaling compared to CBP/HIF-2α.

## References

[1] Chen R, Xu M, Nagati JS, Hogg RT, Das A, Gerad RD, et al (2015) The Acetate/ACSS2 Switch Regulates HIF-2 Stress Signaling in the Tumor Cell Microenvironment. PLoS ONE 10(2): e0116515 doi:10.1371/journal.pone.0116515 25689462 10.1371/journal.pone.0116515 PMC4331492

