# Supplementary figures and images for: The Acetate/ACSS2 Switch Regulates HIF-2 Stress Signaling in the Tumor Cell Microenvironment
Source: PLoS One. 2015 Feb 17;10(2):e0116515. doi: 10.1371/journal.pone.0116515 (PMC4331492; doi:10.1371/journal.pone.0116515)

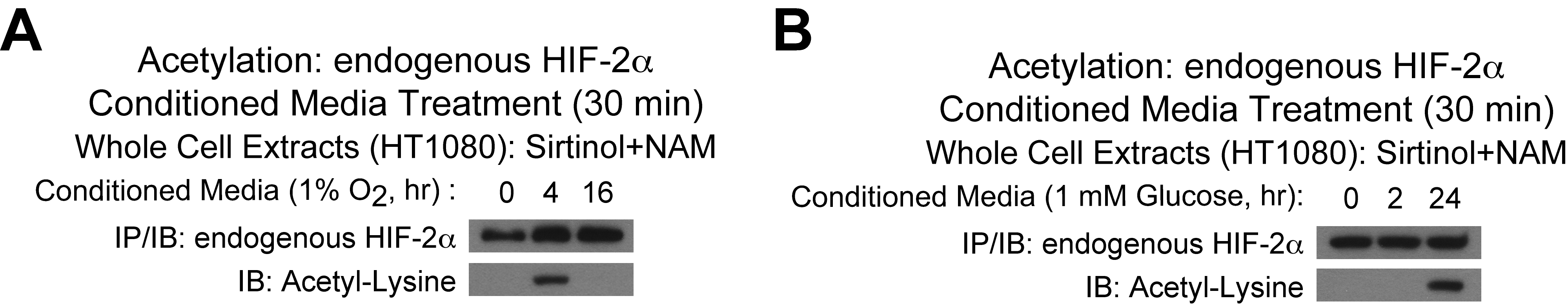

Supplement: S1 Fig — (A) Endogenous HIF-2α acetylation detected by immunoblotting (IB) after immunoprecipitation (IP) and following early (4 hr) or late (16 hr) hypoxia exposure with pharmacological inhibition of Sirt1 by sirtinol and nicotinamide (NAM). (B) Same as (A) except after early (2 hr) and late (24 hr) low glucose exposure. (TIF) [file pone.0116515.s001.tif]

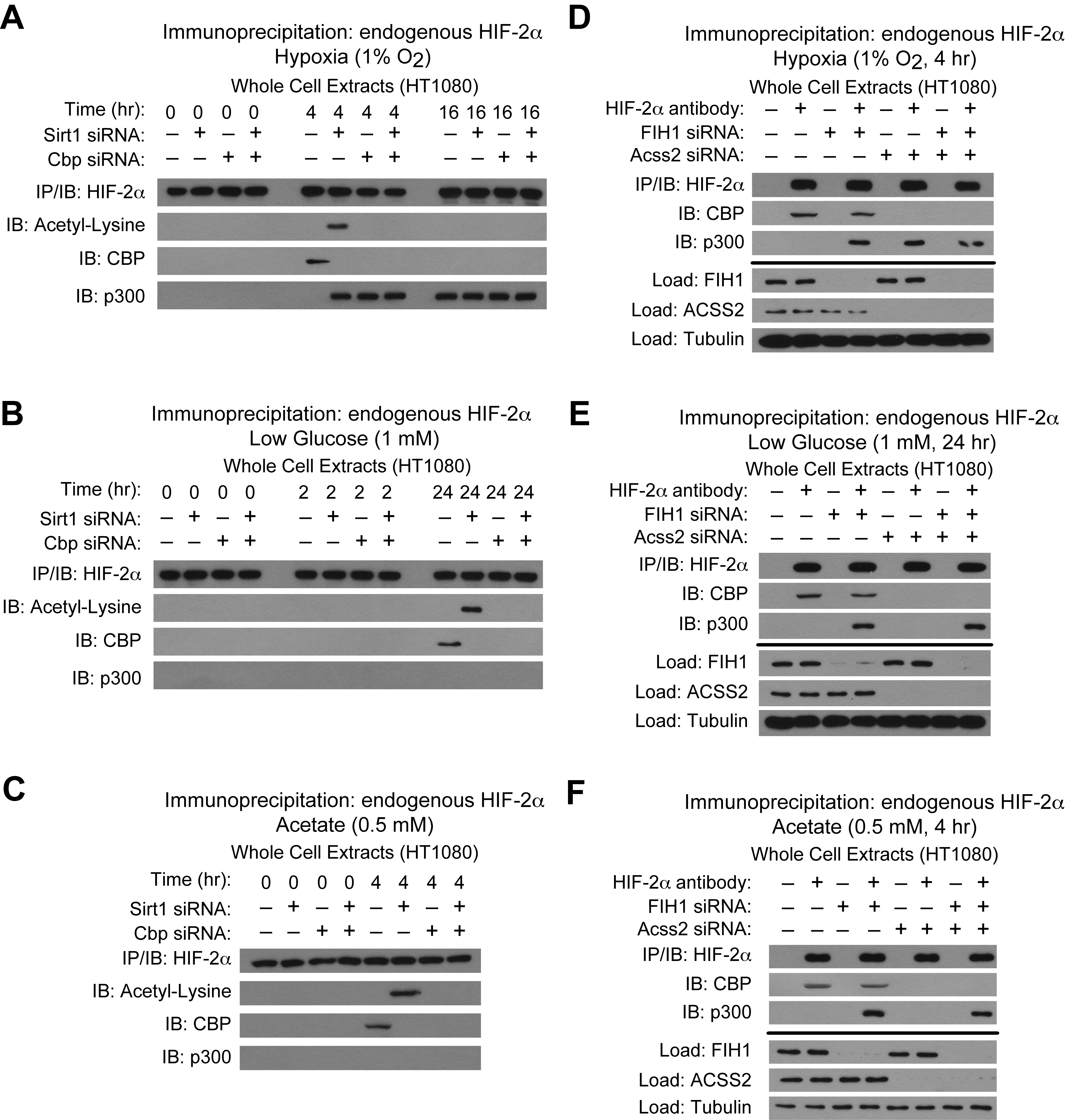

Supplement: S2 Fig — (A) Endogenous CBP/HIF-2α or p300/HIF-2α complexes detected by immunoblotting (IB) after early (4 hr) or late (16 hr) hypoxia exposure following SIRT1, CBP, or combined SIRT1/CBP knockdown. (B) Same as (A) except after early (2 hr) and late (24 hr) low glucose exposure. (C) Same as (A) except after (4 hr) acetate exposure. (D) Endogenous CBP/HIF-2α or p300/HIF-2α complexes after early (4 hr) hypoxia exposure following FIH1, ACSS2, or combined FIH1/ACSS2 knockdown. (E) Same as (D) except after late (24 hr) low glucose exposure. (F) Same as (D) except after (4 hr) acetate exposure. (TIF) [file pone.0116515.s002.tif]

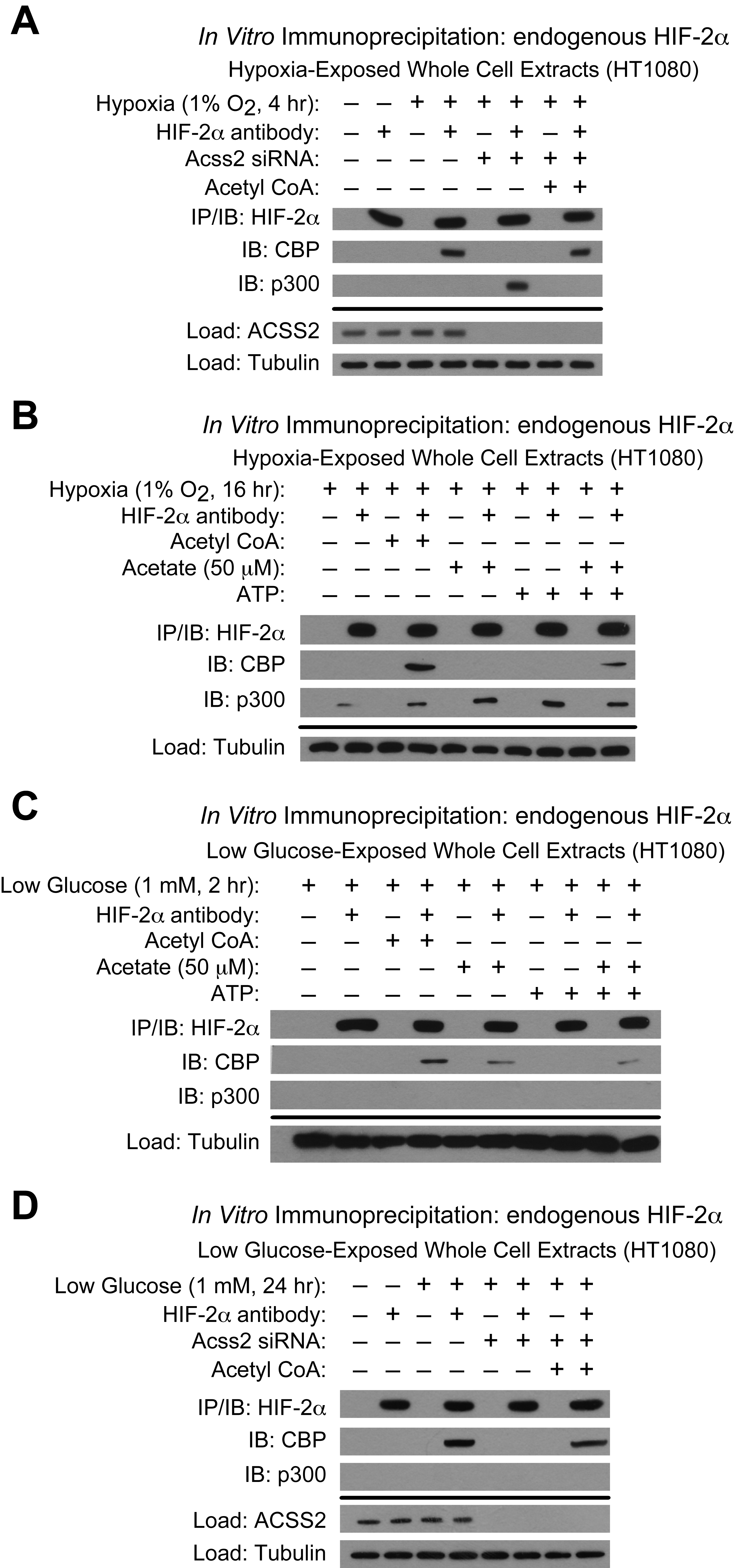

Supplement: S3 Fig — (A) Endogenous CBP/HIF-2α or p300/HIF-2α complexes induced by acetyl CoA addition to HT1080 whole cell extracts prepared following ACSS2 knockdown and 4 hr hypoxia exposure. (B) Endogenous CBP/HIF-2α or p300/HIF-2α complexes induced by acetyl CoA, acetate, ATP, or acetate plus ATP addition to HT1080 whole cell extracts prepared after 16 hr hypoxia exposure. (C) Endogenous CBP/HIF-2α or p300/HIF-2α complexes induced by acetyl CoA, acetate, ATP, or acetate plus ATP addition to HT1080 whole cell extracts prepared after 2 hr low glucose exposure. (D) Endogenous CBP/HIF-2α or p300/HIF-2α complexes induced by acetyl CoA addition to HT1080 whole cell extracts prepared following ACSS2 knockdown and 24 hr low glucose exposure. (TIF) [file pone.0116515.s003.tif]

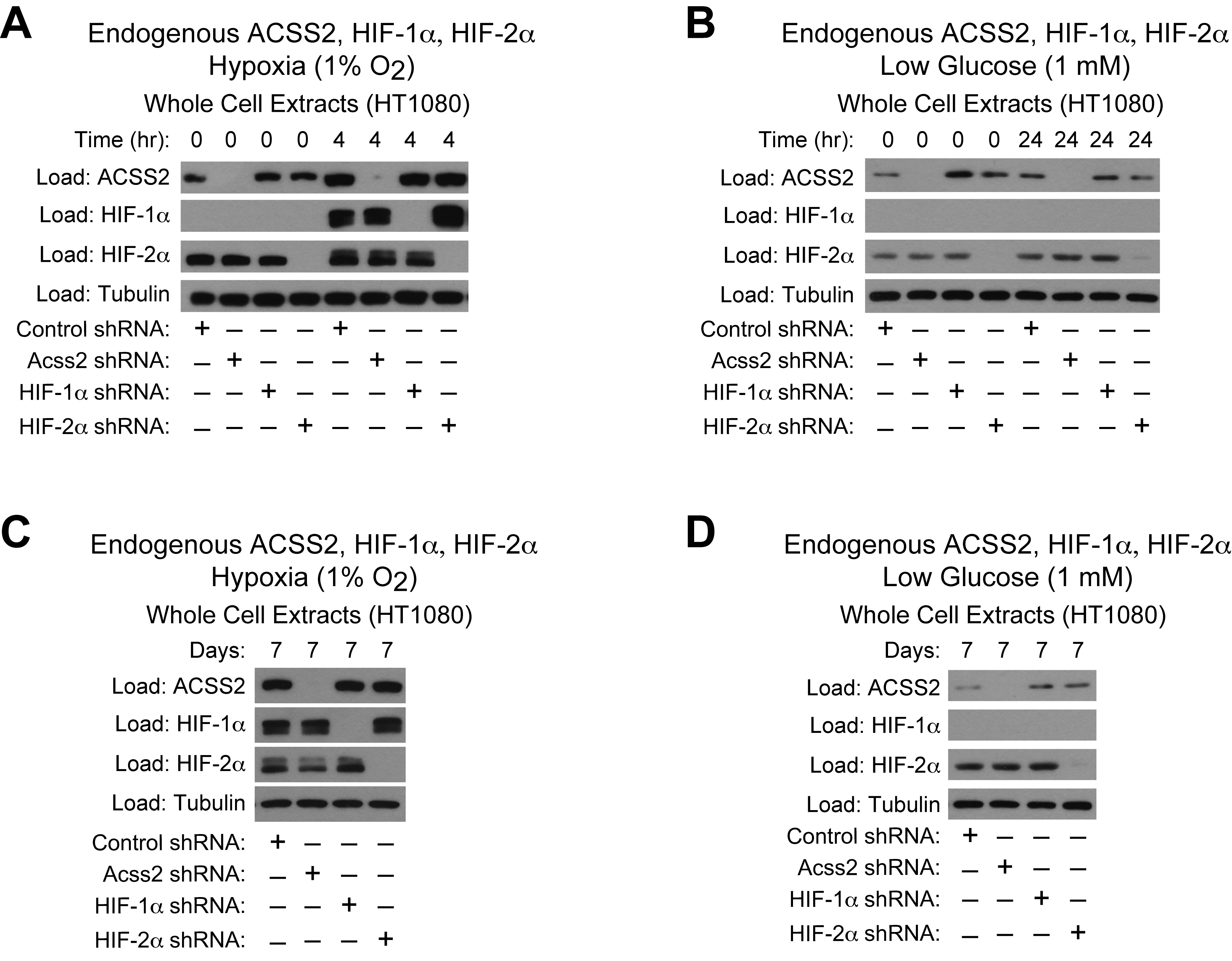

Supplement: S4 Fig — ACSS2, HIF-1α, or HIF-2α protein levels in the indicated stable knockdown cell line following (A) short-term hypoxia (4 hr), (B) short-term glucose deprivation (24 hr), (C) long-term hypoxia (7 day) or (D) long-term glucose deprivation (7 day) stress conditions. HIF-1α protein levels are markedly induced during hypoxia, but are virtually undetectable under basal or low glucose conditions. ACSS2 and HIF-2α protein levels are detectable under all conditions and increase modestly during hypoxia, but not during low glucose condtions. Knockdown of ACSS2, HIF-1α (where detected), or HIF-2α protein in the respective cell line was efficient regardless of either the nature or duration of stress exposure. All experiments performed with HT1080 whole cell extracts. (TIF) [file pone.0116515.s004.tif]

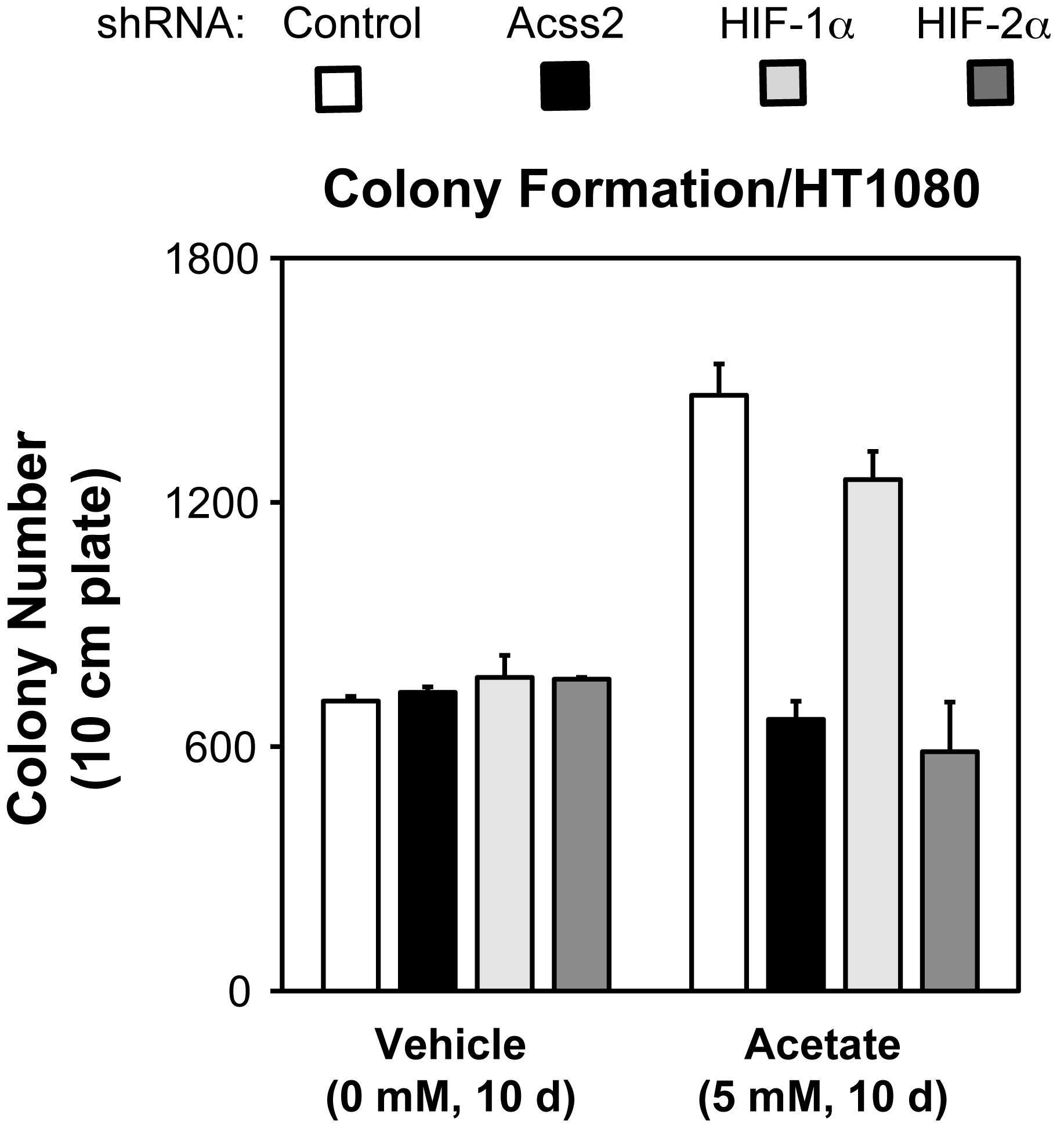

Supplement: S5 Fig — Colony formation for stably transformed HT1080 cells expressing control (white bars), ACSS2 (black bars), HIF-1α (light gray bars), or HIF-2α (dark gray bars) shRNA following (10 d) acetate exposure. Comparison by one-tailed t-test between control and specified knockdown/treatment with significant reductions compared to control indicated (n = 3/treatment; mean/SD; *, P<0.05). (TIF) [file pone.0116515.s005.tif]

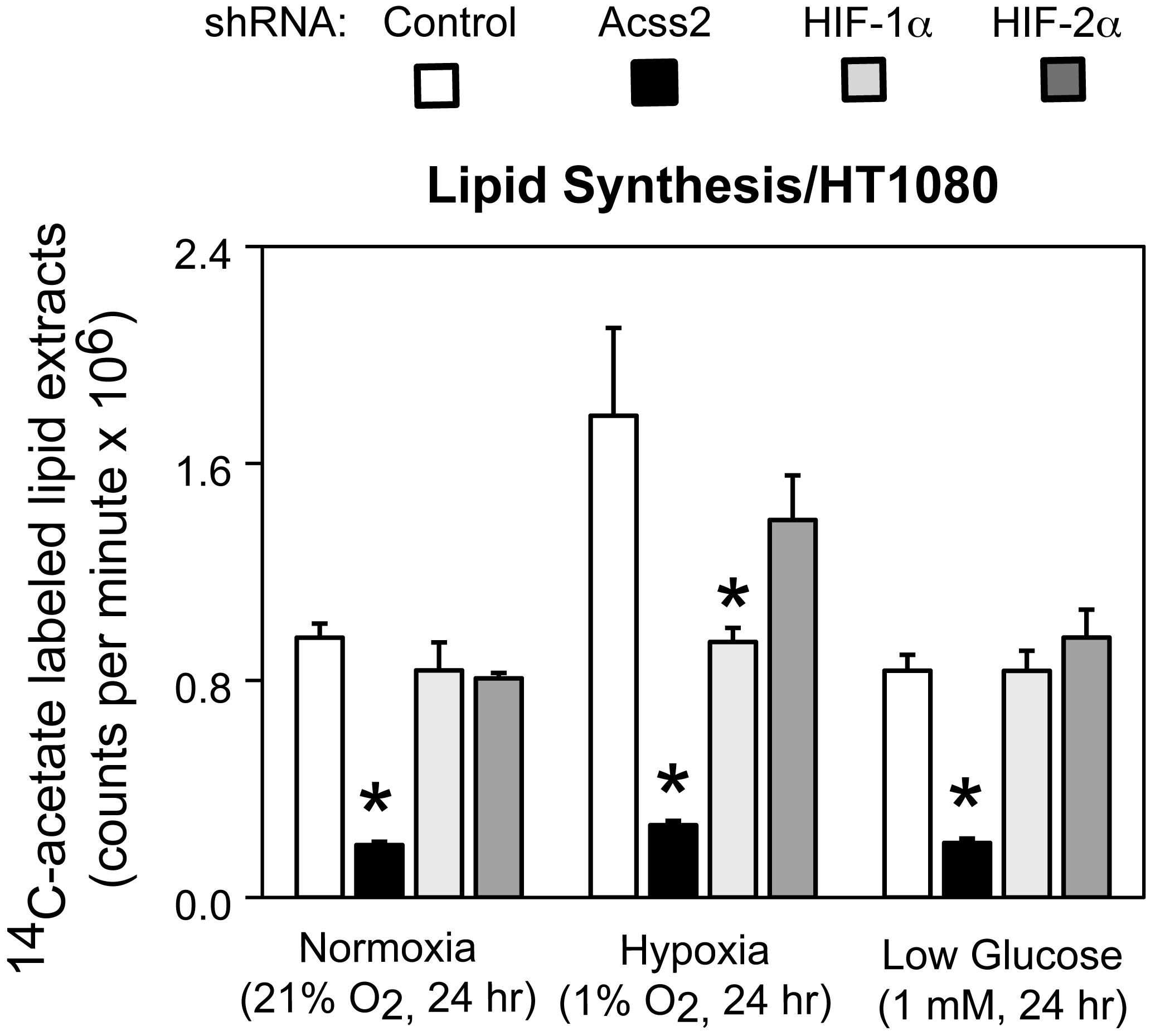

Supplement: S6 Fig — Lipid synthesis as measured by 14C-acetate incorporation in stably transformed HT1080 cells expressing control (white bars), ACSS2 (black bars), HIF-1α (light gray bars), or HIF-2α (dark gray bars) shRNA following (24 hr) normoxia, hypoxia, or low glucose exposure. Comparison by one-tailed t-test between control knockdown/treatment and specified knockdown/treatment with significant reductions compared to control indicated (n = 3/treatment; mean/SD; *, P<0.05). (TIF) [file pone.0116515.s006.tif]
